# Health-related quality of life in patients receiving medicinal cannabis: systematic review and meta-analysis of primary research findings 2015–2025

**DOI:** 10.1007/s11136-026-04170-7

**Published:** 2026-02-01

**Authors:** Margaret-Ann Tait, Louise Acret, Daniel S. J. Costa, Rachel Campbell, Kate White, Claudia Rutherford

**Affiliations:** 1 Faculty of Medicine and Health, Susan Wakil School of Nursing, The University of Sydney, Sydney, NSW Australia; 2 Sydney Local Health District, Sydney, NSW Australia; 3 The Daffodil Centre, The University of Sydney, and Cancer Council NSW, Sydney, NSW Australia; 4 Faculty of Science, School of Psychology, The University of Sydney, Sydney, NSW Australia

**Keywords:** Health-related quality of life, Medicinal cannabis, Patient-reported outcomes, Symptom management, Patient-reported outcome measures, Meta-analysis

## Abstract

**Purpose:**

The global burden of chronic health conditions is significant. Medicinal cannabis (MC) is a legalised treatment option for patients with chronic health conditions in some countries. Health-related quality of life (HRQL) is an important patient-reported outcome across all chronic health conditions. We aimed to determine how studies of MC therapy justify, measure, and report HRQL, and assess the current evidence for HRQL following MC treatment.

**Methods:**

Systematic review searching AMED, Medline, Web of Science, Scopus, Embase, Cinahl, and PsycINFO from Jan 2015 to Apr 2025. Studies using validated HRQL measures pre-, and post-MC treatment for any chronic health condition were included. Screening and data extraction were performed independently by two reviewers. Completeness of HRQL reporting was evaluated. Meta-analyses for short-term (2-weeks to 3-months), medium-term (> 3 to < 12-months), and long-term (≥ 12-months) HRQL outcomes were conducted, with Risk of Bias (RoB) assessed in randomised control trials (RCTs).

**Results:**

Of 16,674 retrieved citations, 64 studies were retained for analysis:12 RCTs; 38 cohort studies; 13 case series; 1 non-randomised experimental study. Thirty-nine studies (61%) provided justification for assessing HRQL and five (8%) provided HRQL definitions. Studies used generic (*n* = 52, 81%) or condition-specific (*n* = 12, 19%) HRQL measures, with EQ-5D-5L most commonly used. Meta-analyses: RCTs showed small short-term HRQL improvements (Cohen’s d = 0.30, *p* = 0.03), with some concerns or low RoB. For observational studies, HRQL improved in all follow-up periods (d = 0.43 to 0.74; all *p* < 0.001). HRQL improvement varied between, and within, different health conditions.

**Conclusion:**

This systematic review and meta-analyses of studies published between 2015 and 2025 found that few studies provided HRQL definitions, and a third of studies did not explain why they measured HRQL. To ensure appropriate measures are used for this important treatment outcome, future studies should define HRQL and justify the HRQL assessment in the context of research objectives. Overall, improvements in HRQL were observed across studies of patients using MC.

**Supplementary Information:**

The online version contains supplementary material available at 10.1007/s11136-026-04170-7.

## Abstract (plain language summary)

Medicinal cannabis (MC) is a treatment option for patients in countries where prescribing MC is legal. Health-related Quality of Life (HRQL) can mean different things to different people but remains an important treatment outcome for all patients, regardless of their specific health condition. HRQL varies depending on the context and measurement tool (questionnaire) used. We aimed to find out (1) how HRQL assessment is justified, defined and measured in MC research, and (2) if HRQL improves in patients prescribed MC. We looked at studies published over the past decade that reported HRQL in patients before and after MC treatment. Most studies (81%) used generic HRQL questionnaires (e.g., EuroQol Group: EQ-5D) and others used condition-specific questionnaires (e.g. Multiple Sclerosis Quality of Life: MSQoL-52). Overall, HRQL improved in patients using MC. However, only a few studies provided HRQL definitions, and a third of studies didn’t explain why they measured HRQL. This information is needed to ensure HRQL is measured and interpreted appropriately in future studies.

**Supplementary Information:**

The online version contains supplementary material available at 10.1007/s11136-026-04170-7.

## Introduction

Globally, chronic health conditions place a significant burden on individuals, health systems, and society more broadly, with chronic pain being the leading cause of disability worldwide [1]. The identification of cannabinoids, cannabidiol (CBD) and Δ9-tetrahydrocannabinol (THC), as analgesics in 1940 ignited interest in using a cannabis-based medicine as adjunctive treatment for the symptomatic relief of chronic pain [2]. However, most United Nations countries criminalised the consumption of cannabis in the 1960’s [3], consequently limiting research in this area. The past few decades have seen a resurgence of research on cannabis therapy, particularly after the discovery of the endocannabinoid system in the 1990’s and its important role in regulating mood and emotional responses [4]. Medicinal Cannabis (MC) began to be posited as a potential alternative to opioids associated with increasing incidence of adverse events [5], including hospitalization and death from overdose [6]. To enable MC research and build an evidence base for its therapeutic efficacy [7], many countries have since revised their policies and introduced legislation to allow the use of cannabis for research and medicinal purposes, starting with Canada in 2001 [8], and most recently, Ukraine in 2024 [9].

### Patient-reported outcomes and the importance of health-related quality of life

A patient-reported outcome (PRO) is any report coming directly from patients, without interpretation by physicians or others, about how the patient feels in relation to their health condition and its therapy [10]. PROs are particularly important endpoints to include in clinical studies of patients with chronic conditions where the primary aim is to palliate symptoms [11]. A validated PRO measure (PROM) is a standardised questionnaire that allows comparisons between treatment groups, and within groups and individuals over time. PROMs can measure symptom burden, aspects of functioning, and health-related quality of life (HRQL). In this review, HRQL is defined as a “*multidimensional construct encompassing perceptions of the impacts – positive and negative - of a disease or its treatment on physical*,* emotional*,* social*,* and cognitive functions*,* as well as somatic discomfort and other symptoms*” [12]. There are other definitions of HRQL, for example, “*the value assigned to the duration of survival as modified by the impairment*,* functional states*,* perception and social opportunities that are influenced by disease*,* injury*,* treatment*,* or policy*” [13]. Optimal HRQL is universally valued as an important outcome across all health conditions, making it an important endpoint in clinical research. Considering the various ways of conceptualising HRQL, definitions will depend on the research context. It is important for researchers to be aware that HRQL is often defined, measured, and reported in different ways, and to be explicit about the specific meaning and context of HRQL in their studies [14]. Having a clear definition helps inform the selection of appropriate HRQL measures and improve scientific rigor. This is particularly important considering HRQL results can differ within the same group of patients depending on the PROM used to measure HRQL [14].

### Previous HRQL evidence for MC

Two systematic reviews of HRQL in patients treated with MC have been published covering studies from inception to April 2015: Goldenberg et al. 2017 [15], and Whiting et al. 2015 [16]. Between them, they looked at 14 conditions including chemotherapy-induced nausea and vomiting, chronic pain, multiple sclerosis spasticity, depression, inflammatory bowel disease, anxiety, and sleep disorder. Goldenberg et al. found small HRQL improvements in patients with pain, multiple sclerosis, and inflammatory bowel disease [15]. Moderate-quality evidence supported MC for treating chronic pain and spasticity, but only low-quality evidence for chemotherapy-induced nausea and vomiting, appetite, sleep disorders, and Tourette syndrome [16]. In meta-analyses, overall effect sizes (Cohen’s d) were small and non-significant: 0.05 for HRQL [15] compared with controls, and 0.35 for HRQL [15] in pre- and post-therapy observational studies. However, analyses included all available data without categorising results by follow-up period. For example, HRQL assessed two weeks after MC therapy was combined with studies reporting HRQL following many months of therapy [15]. Notably, half of studies included within both reviews used synthetic cannabinoids rather than cannabinoids extracted from the cannabis plant (presumably due to historical legal and access restrictions). Since then, more countries have supported MC research and production, and non-synthetic MC formulations, and methods of administration have been further refined. Furthermore, access to MC often relies on the judgement and clinical justification of a prescribing doctor, which may cover a wider range of health conditions than previously reviewed.

### Objectives and research aims

This review analysed all new evidence since 2015 assessing patients’ HRQL before and after receiving MC treatment. The research aims were to: (1) determine how HRQL was defined in studies of patients being prescribed MC, and identify which PROMs were used for HRQL assessment and the rationale for choosing them; (2) assess the quality and completeness of HRQL reporting in MC research; and (3) through meta-analyses, determine any short-, medium-, and long-term HRQL improvements in patients receiving MC.

## Methods

### Literature searches

This systematic review was prospectively registered with PROSPERO (CRD42022306778) [17] and followed guidelines from Preferred Reporting Items for Systematic Reviews and Meta-Analyses (PRISMA) [18]. Bibliographic databases were searched (MEDLINE, Web of Science, PsycINFO, EMBASE, AMED - Allied and Complimentary Medicine, Scopus, and Cinahl) for studies published since 1 January 2015 until 8 April 2025 using MeSH terms “medical marijuana” or “Cannabis” and “quality of life” or “patient-reported outcome measures”. See Online Resource 1 for example of search strategy used in MEDLINE. Electronic searches were supplemented by a search of reference lists of eligible studies.

### Inclusion criteria

Two reviewers screened studies independently. Randomised and non-randomised controlled trials and longitudinal observational studies with pre-treatment baseline, including cohort studies and case series were included. Only studies of adults (over 18 years) with any diagnosed health condition using MC (prescribed or approved by a medical practitioner) for symptom management, using validated generic or disease-specific multi-item PROMs to assess HRQL at baseline (before therapy) and follow-up (greater than 2-weeks) were included. MC was defined as a non-synthetic pharmaceutical cannabinoid product. Cannabinoid products may constitute one or more of any cannabinoid, including the major cannabinoids; delta-9-tetrahydrocannabinol (THC), and cannabidiol (CBD), and/or minor cannabinoids, for example, cannabichromene (CBC), cannabidivarin (CBDV), cannabichromevarin (CBCV), cannabigerol monomethyl ether (CBGM), cannabinol (CBN), cannabitriol (CBT), tetrahydrocannabutol (THCB), and tetrahydrocannabiorcol (THCC), and many more. Randomised and non-randomised clinical trial comparators included usual care, placebo or no treatment. Observational cohort and case series did not require a control group.

Studies with children and adolescents under 18 years, adults unable to self-report (i.e. assessments by proxy), cross-sectional studies, healthy participants, studies looking at synthetic cannabinoids, and studies not assessing HRQL with validated PROMs were excluded.

Published studies were limited to English language, full text (not conference abstracts), and primary research (not reviews, systematic reviews, meta-analyses, or commentary).

### Outcomes

Results for overall HRQL were extracted according to predefined time periods: short-term (2 weeks to 3-months), medium term (between 3-months and 12-months), and long-term (12-months or longer), following Cochrane guidelines for systematic reviews in back pain, [19] and time periods reported in similar reviews of chronic health interventions [20, 21]. As patient treatment typically involves a titration period, acute HRQL assessments (within 2 weeks of commencing treatment) were excluded [22]. Changes of patient-reported HRQL from baseline to last available follow-up within the predefined time periods were extracted.

### Data extraction

Standardised data extraction forms developed on the Covidence platform [23] were piloted on a small sample of papers (*n* = 5) and adapted as necessary. To minimise bias and errors, data extraction involved two independent reviewers. Disagreements were resolved through discussion until consensus was reached or referred to a third reviewer where necessary.

The following information was extracted from included studies: First author; year of publication; country; study design; aim; sample size; health condition(s); MC treatment type; comparison group; HRQL definitions; justification for measuring HRQL; HRQL PROM; assessment timepoints; HRQL results (means; medians; standard deviations; IQR; effect sizes; confidence intervals; statistical tests; odds ratios); and study limitations.

### Quality assessment

The completeness of reporting for included studies was scored formally by two reviewers independently following criteria outlined in the STROBE Checklist for reporting observational studies [24], and CONSORT-PRO checklist for reporting PROs in randomised controlled trials (RCT) [25], using data extraction templates managed on the Covidence platform [23]. Existing tools for completeness of reporting quantitative clinical study designs inadequately address key issues for PRO assessment. Therefore, this review assessed the completeness of PRO reporting, specifically. The quality of completeness assessment data extraction template combined CONSORT and STROBE items of similar content and integrated the 14 CONSORT-PRO extension items. To standardise scores across both RCTs and observational studies, items that were RCT-specific were replaced with corresponding STROBE items for observational studies (e.g. CONSORT items 20–22 regarding discussion topics, were replaced with STROBE items 19–21). As typically done with the CONSORT and STROBE checklists, total reporting completeness scores using the revised PRO reporting checklist were calculated as a percentage of total possible score. The final revised PRO reporting checklist of 18 items and criteria are included in Online Resource 2. Additionally, Risk of Bias (RoB) was assessed separately for RCTs included in the meta-analysis using the revised Cochrane Tool; RoB 2, designed specifically for RCTs and for assisting the interpretation of meta-analyses [26].

PRO reporting completeness and RoB 2 was scored by two reviewers independently. Each criterion was scored, and inter-assessor consistency cross-checked with discrepancies discussed until consensus reached.

### Analysis

A narrative summary included study characteristics, i.e., study design and aims, population size, geographical location, year, baseline population characteristics, HRQL definition, justification for assessment and PROMs used. Where data was not reported in a format suitable for pooling (e.g. missing measures of variability) or datasets unavailable, study findings were synthesised using descriptive text and tables. Meta-analyses were run separately for studies reporting between group differences and within group differences and for different follow-up time periods (short-, medium-, and long-term). Where multiple studies were conducted on the same participants, analyses for each follow-up time period included data from one report only, selected by highest participant number. A random-effects model with effect size assumed to have a distribution within the population was used to estimate summary measures of effect. Medians and IQR were converted to means and SD using R package (estmeansd) [27]. For within group analyses, estimates of standard deviation of difference scores and standard error of effect sizes assumed a correlation between repeated measures of *r* = 0.5 [28]. In studies reporting HRQL from multiple PROMs and/or scales, results from one total or utility score were used where possible, chosen as the most comparable with those reported by other included studies. When multiple domain scores were reported instead of a single HRQL score, previously established domain correlations were used when calculating overall effect size of study (e.g. *r* = 0.62 for SF-36 physical and mental composite scores) [29]. To reduce possible bias from erroneous data, we crossed-checked all statistical information available for determining study effect sizes, including t tests and reported p-values, and where relevant, chose the most conservative estimation for inclusion in meta-analyses. Effect size (Cohen’s d) and variation of effect size were calculated in Microsoft Excel spreadsheets, and meta-analyses conducted with IBM SPSS Statistics 28.0 program. As a sensitivity analysis, meta-analyses with high heterogeneity (*τ*^2^ > 0.1) were re-run without outliers (Cohen’s d > 1.5) and reported separately.

## Results

**Fig. 1:**
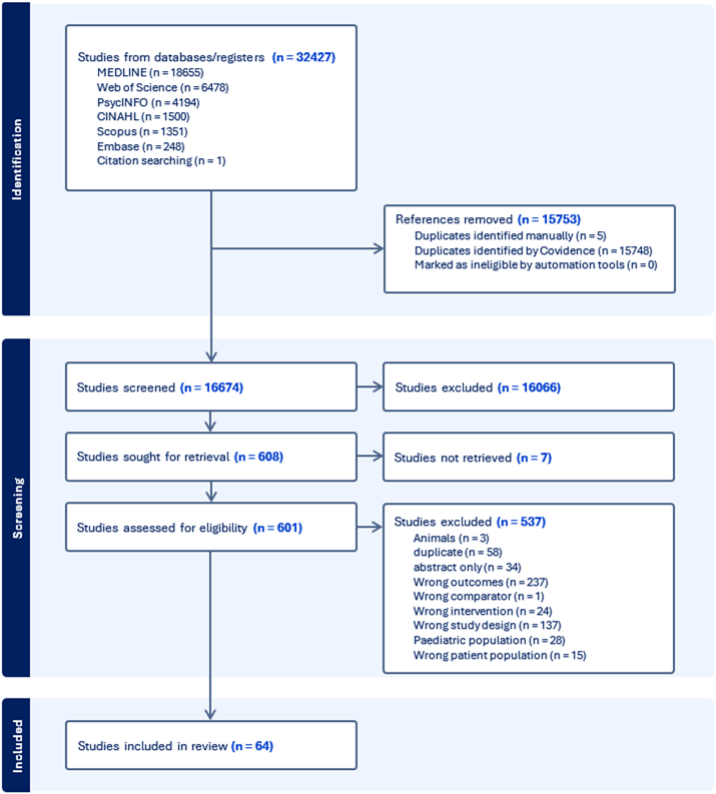
PRISMA flow diagram of studies through the review process

**Fig. 2:**
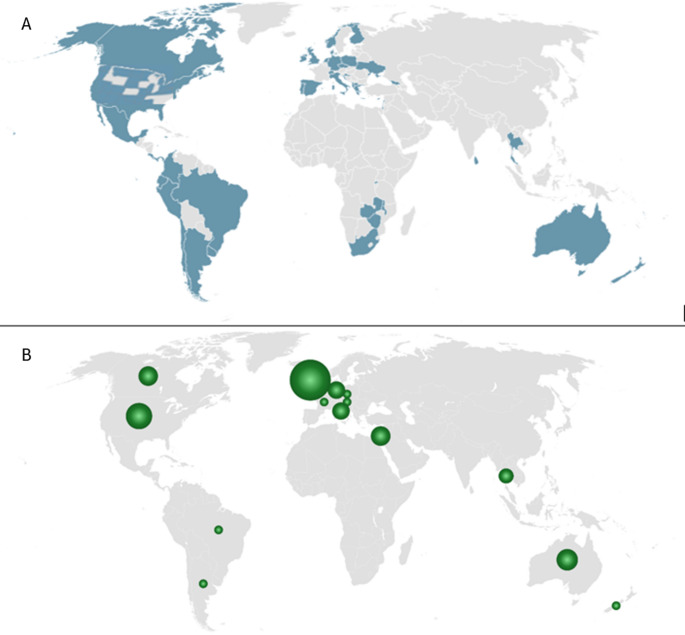
**A** Countries and regions where MC is legal and **B** Bubble plot distribution of studies assessing HRQL in MC patients globally

The search yielded 16,674 studies minus duplications. Title, abstract and full text screening resulted in 64 studies of 43,847 patient participants included in our analysis. Results from the search are summarized in the PRISMA diagram (Fig. 1). Most studies were observational cohort studies (*n* = 38, 59.4%), with the remaining being case series (*n* = 13, 20.3%), RCTs using parallel (*n* = 9, 14.1%) or crossover (*n* = 3, 4.7%) designs, and one non-randomised experimental study (1.5%). An overview of included studies is provided in Online Resource 3. The number of studies conducted in each country is represented in Fig. 2 alongside a map of countries and regions where MC is legal. Apart from south African regions and Sri Lanka, the distribution of studies was largely representative of countries where MC is legal. A third of included studies were conducted in the United Kingdom (*n* = 22, 34%), with 19 collecting data through the United Kingdom Medical Cannabis Registry. The health conditions of participant groups studied included Chronic Pain (*n* = 12) [6, 30–40], Multiple Sclerosis (*n* = 8) [41–48], Cancer (*n* = 5) [49–53], Anxiety disorder (*n* = 4) [54–57], Inflammatory Bowel Disease (includes Crohn’s disease and Ulcerative colitis) (*n* = 4) [58–61], Epilepsy (*n* = 3) [62–64], Fibromyalgia (*n* = 3) [65–67], Arthritis (*n* = 2) [68, 69], Sleep disorder (*n* = 2) [70, 71], Chronic neuropathic pain (*n* = 2) [72], HIV (*n* = 2) [72, 73], Post Traumatic Stress Disorder (*n* = 1) [74], Primary Headache Disorder (*n* = 1) [75], Hypertension (*n* = 1) [76], or any condition eligible for MC (*n* = 16) [77–91]. Online Resource 4 lists health conditions of all participants treated with MC in this review (including studies recruiting patients with any condition eligible for MC).

### How HRQL is defined, justified and evaluated

Although 51 of the 64 studies (80%) mentioned HRQL (or quality of life) in the title or abstract, and 34 (53%) reported HRQL as their primary outcome, only five (8%) studies provided a definition of HRQL (Table 1) [38, 76, 80, 83, 85]. References for HRQL definitions were provided by four of the five studies and sourced from Osoba et al. [12] and the US Centres for Disease Control and Prevention [92]. A reason or justification for including HRQL as an endpoint was provided in 39 (61%) studies. PROMs used to assess HRQL and the methods for reporting HRQL varied across studies. Most studies (*n* = 51; 80%) provided references for PROMs (Table 1). Of the 52 (81%) studies using generic HRQL PROMS, the most frequently used was the EuroQol Group EQ-5D-5L [93], (*n* = 31, 48%) or earlier version EQ-5D-3 L (*n* = 1, 1.5%), reporting five domain scores and a single utility index (*n* = 24, 37%), score from the visual analogue scale presented at the end of the PROM (*n* = 5, 8%), or five domain scores only (*n* = 2, 3%). Another frequently used generic PROM was the RAND SF-36 [94] (*n* = 14, 22%) or shorter version, SF-12 (*n* = 2, 3%), typically reporting two composite summary scores, physical and psychological (*n* = 6, 9%), 8 domain scores (*n* = 7, 11%), or a total score (*n* = 2, 3%) (Table 1). Twelve studies (19%) used condition-specific HRQL PROMs; most frequently, the Multiple Sclerosis Quality of Life [95], (MSQoL-54; *n* = 3, 5%); and variants of the Quality of Life in Epilepsy scale [96], QOLIE-89, QOLIE-31P, and QOLIE-10 (*n* = 3) (Table 1). One study utilised four PROMs covering pain, sleep, and mood and collectively referred to them as measuring HRQL [40]. Table 1 displays characteristics of all included studies and a summary of significant or clinically important PRO findings.

**Table 1:** Study characteristics, HRQL reporting, and summary of findings of included studies

References	Health condition	MC composition	Total *N* (% male)	Mean age (SD or range)	Follow-up [Recruitment dates]	Study design	HRQL assessment justified?	HRQL definition? [citation?]	HRQL PROM(s) [citation?]	Summary of HRQL findings
Aiewtrakoon et al. [71]	Insomnia	CBD	45 (33)	45.96 (11.7)	2-w [Sep 2021–Apr 2023]	RCT - crossover	No	No	EQ-5D-5L [no]	Improved HRQL compared with placebo at 2-weeks (index score)
Arkell et al. [77]	Any condition	THC: CBD	3148 (46.4)	55.9 (18.7)	20-m [Dec 2018–May 2022]	Case series	Yes	No	SF-36 [yes]	Improved HRQL at 12-months (across all 8 domains).
Aungsumart et al. [46]	Multiple Sclerosis spasticity	THC: CBD	7 (42.8)	41.3 (1.8)	12-w [Nov 2019–Jun 2020]	Cohort study	Yes	No	EQ-5D-5L [no]	No change in HRQL at 12-weeks (index score)
^‡^Bapir et al. [34]	Chronic Pain	CBD/THC/ THC: CBD	1254 (54.1)	45.7 (14.4)	3-m 6-m [Dec 2021–Jan 2022]	Cohort study	Yes	No	EQ-5D-5L [yes]	Improved HRQL at 3- and 6-months (index score)
Barre et al. [73]	HIV	CBD	79 (69.6)	55.97 (12.8)	12-w [May 2022–Oct 2022]	RCT - parallel	Yes	No	SF-36 [yes]	No change in HRQL compared with placebo at 12-weeks (physical and mental composite summary scores). Of 8 domains, only physical functioning improved
Bar-Sela et al. [50]	Cancer	THC: CBD	34 (41)	63 (nr)	3-m [‘*2 years*’]	Non-randomised experimental	No	No	QLQ-30 [yes]	No change in HRQL at 3- months (global QoL). Improved insomnia and appetite (QLQ-C30 domains)
Brett et al. [62]	Epilepsy	CBD	17 (50)	34.6 (18–64)	2.5-m 5-m [Aug 2017–Jun 2019]	Cohort study	Yes	No	QOLIE-31P [yes]	Improved HRQL at 5-months (total score)
Capano et al. [40]	Chronic Pain	CBD	97 (32)	56.2 (39–70)	8-w [Sep 2018–Dec 2018]	Cohort study	No	No	PHQ-4, PDI, PSQI, PEG [no]	Improved HRQL at 8-weeks (sleep and pain PROMs only). No improvement in PHQ-4 or PDI
Chaves et al. [67]	Fibromyalgia	THC	18 (0)	51.9 (nr)	8-w [Sep 2019–Nov 2019]	RCT - parallel	Yes	No	FIQ [yes]	Improved HRQL compared with placebo at 8-weeks (total score)
^‡^Datta et al. [33]	Chronic Pain	CBD/THC/ THC: CBD	1139 (54)	47 (14.1)	3-m 6-m 12-m [Dec 2019–Jan 2023]	Cohort study	Yes	No	EQ-5D-5L [yes]	Improved HRQL at 3-, 6- and 12-months (index score)
^‡^Dickinson et al. [30]	Chronic Pain (Hypermobility-associated)	THC: CBD	161 (19.3)	37.42 (10.5)	3-m 6-m 18-m [Dec 2019–Dec 2023]	Case series	No	No	EQ-5D-5L [yes]	Improved HRQL at 3-, 6- and 18-months (index score)
Dujic et al. [76]	Hypertension	CBD	64 (57.8)	54.4 (8.9)	5-w [Dec 2021–Apr 2022]	RCT - crossover	Yes	Yes [No]	SF-36 [yes]	No change in HRQL compared with placebo at 5-weeks (VAS presented after SF-36). Of 8 domains, only fatigue and psychological well-being improved
Eibach et al. [72]	HIV-Associated Neuropathic Pain	CBDV	32 (97)	50.31 (9)	3-w [Jan 2017–Jan 2019]	RCT - crossover	Yes	No	SF-36 [yes]	No change in HRQL compared with placebo at 3-weeks (across all 8 domains).
^†^Ergisi et al. [78]	Any condition	THC: CBD	312 (55.1)	44.8 (15.4)	3-m 6-m [Dec 2019–nr]	Case series	Yes	No	EQ-5D-5L [no]	Improved HRQL at 3- and 6-months (index score)
^†^Erridge et al. [79]	Any condition	THC: CBD	129 (51.2)	46.23 (14.5)	3-m [Dec 2019–nr]	Case series	Yes	No	EQ-5D-5L [yes]	Improved HRQL at 3-months (VAS)
^‡^Francis et al. [68]	Inflammatory Arthritis-associated Chronic Pain	THC: CBD	82 (50)	47.61 (14.3)	3-m 6-m 12-m [Dec 2019–Jan 2023]	Case series	No	No	EQ-5D-5L [yes]	Improved HRQL at 3-, 6- and 12-months (index score)
^‡^Francis et al. [69]	Osteoarthritis-related Chronic Pain	THC: CBD	77 (52)	60.04 (14.3)	3-m 6-m 12-m [Dec 2019–Jan 2022]	Case series	Yes	No	EQ-5D-5L [yes]	Improved HRQL at 3- and 6-months (index score)
Gaston et al. [64]	Epilepsy	CBD	53 (50.9)	nr	12-m [nr]	Cohort study	Yes	No	QOLIE-89 [yes]	Improved HRQL at 12-months (total score).
Gruber et al. [91]	Any condition	THC: CBD	11 (55)	48.91 (15.1)	3-m [nr]	Cohort study	No	No	SF-36 [yes]	Improved HRQL at 3-months (fatigue domain only)
Gruber et al. [39]	Chronic Pain	THC: CBD	37 (44)	54.87 (14.0)	3-m 6-m [nr]	Cohort study	Yes	No	SF-36 [yes]	Improved HRQL at 3- and 6-months (fatigue, pain and general health domains only)
Gulbransen et al. [90]	Any condition	CBD	397 (46.1)	51.48 (19.1)	3-w [Dec 2017–Dec 2018]	Cohort study	No	No	EQ-5D-5L [yes]	Improved HRQL at 3-weeks (VAS)
Gupta et al. [58]	Inflammatory Bowel Disease	THC: CBD	116 (81)	39 (9.1)	3-m 6-m 18-m [Dec 2019–Dec 2023]	Case series	Yes	No	EQ-5D-5L [yes]	Improved HRQL at 3-, 6- and 18-months (index score)
Hardy et al. [52]	Advanced Cancer	CBD	144 (52.8)	64.6 (12.8)	28-days [Feb 2019–Nov 2021]	RCT - parallel	No	No	QLQ-C15 [yes]	No change in HRQL compared with placebo at 28-days (global QoL)
Haroutounian et al. [6]	Chronic Pain	THC: CBD	206 (61.7)	51.2 (15.4)	6-m [Jun 2010–Jan 2013]	Cohort study	No	No	S-TOPS [yes]	Improved HRQL at 6-months (all except physical disability domains)
Haupts et al. [48]	Multiple Sclerosis spasticity	THC: CBD	241 (42)	49.1	3-m [nr]	RCT - parallel	Yes	No	EQ-5D-5L SF-36 [no]	No change in HRQL compared with placebo at 3-months (EQ-5D index score and across SF-36 domains)
Haupts et al. [42]	Multiple Sclerosis spasticity	THC: CBD	54 (39)	51.4 (11.1)	12-w [Dec 2021–Jan 2023]	Cohort study	No	No	MFHW [yes]	No change in HRQL at 12-weeks (total score)
Hershkovich et al. [66]	Fibromyalgia	THC: CBD	30 (0)	46 (5)	1-month [nr]	Cohort study	Yes	No	WHOQOL-BREF [yes]	Improved HRQL at 1-month (pain, sleep, physical, and psychological domains only)
Irving et al. [61]	Inflammatory Bowel Disease (Ulcerative colitis)	CBD	60 (79)	43.77 (13.9)	10-w [nr]	RCT - parallel	No	No	IBDQ [no]	Improved HRQL compared with placebo at 10-weeks (total score)
^‡^Kawka et al. [32]	Chronic Pain	THC: CBD	110 (49.1)	52.1 (15.4)	3-m 6-m [Dec 2019–nr]	Case series	Yes	No	EQ-5D-5L [yes]	Improved HRQL at 3-months (index score)
Kelley et al. [81]	Any condition	THC: CBD	103 (39.8)	nr (18–65+)	30-days [nr]	Cohort study	Yes	No	SF-36 [no]	Improved HRQL at 30-days (across all domains).
Lamonarca et al. [63]	Epilepsy	CBD	44 (34)	35 (10)	3-m 6-m [nr]	Cohort study	Yes	No	QOLIE-10 [yes]	Improved HRQL at 3- and 6-months (total score)
Lent et al. [83]	Any condition	THC: CBD	438 (33.6)	46.4 (15.6)	3-m [Sep 2020–Jun 2023]	Cohort study	Yes	Yes [Yes]	SF-36 [yes]	Improved HRQL at 3-months (across all domains)
^#^Li et al. [54]	Generalised Anxiety Disorder	THC: CBD	120 (61.7)	39.38 (12.48)	3-m 6-m 12-m [Dec 2019–nr]	Case series	No	No	EQ-5D-5L [yes]	Improved HRQL at 3-, 6- and 12-months (index score)
Lucas et al. [89]	Any condition	CBD/THC/ THC: CBD	1145 (42.4)	51.2 (15.4)	3-m 6-m [nr]	Cohort study	No	No	WHOQOL-BREF [yes]	Improved HRQL at 3- and 6-months (across all 4 domains)
Lynskey et al. [82]	Any condition	THC: CBD	198 (47)	72.2	3-m [Aug 2020–Apr 2023]	Cohort study	yes	no	EQ-5D-5L [yes]	Improved HRQL at 3-months (index score)
Markova et al. [47]	Multiple Sclerosis spasticity	THC: CBD	191 (29.8)	51.3 (10.2)	12-w [nr]	RCT - parallel	Yes	No	SF-36 [no]	No change in HRQL compared with placebo at 12-weeks (general health domain)
Meng et al. [88]	Chronic Pain	THC: CBD	757 (38.3)	nr (18–65+)	3-m 6-m 12-m [Sep 2015–Jul 2018]	Cohort study	Yes	No	EQ-5D-3 L [yes]	Improved HRQL at 3-, 6- and 12-months (EQ-5D VAS)
^#^Murphy et al. [55]	General Anxiety Disorder	THC: CBD	302 (69.5)	38.06 (11.7)	3-m 6-m 12-m [Dec 2019–Jan 2023]	Case series	Yes	No	EQ-5D-5L [yes]	Improved HRQL at 3-, 6- and 12-months (index score)
Murphy et al. [41]	Multiple Sclerosis	THC: CBD	141 (48.9)	45.89 (11.1)	3-m 6-m [Dec 2019–Aug 2022]	Cohort study	Yes	No	EQ-5D-5L MSQoL-54 [yes]	Improved HRQL at 3 and 6 months (EQ-5D index score and MSQoL-54 physical and mental composite summary scores)
Naftali et al. [59]	Inflammatory Bowel Disease (Crohn’s Disease)	THC: CBD	56 (33)	34.5 (11)	8-w [2013–2018]	RCT - parallel	No	No	SF-36 [yes]	Improved HRQL compared with placebo at 8-weeks (total score)
Naftali et al. [60]	Inflammatory Bowel Disease (Ulcerative colitis)	THC	32 (56)	32 (10.86)	8-w [nr]	RCT - parallel	Yes	No	SF-36 [yes]	Improved HRQL compared with placebo at 8-weeks (total score)
Nathan et al. [51]	Cancer	THC: CBD	83 (58)	nr (65–81+)	3-m [Jan 2018–Dec 2018]	Cohort study	No	No	ESAS [no]	No change in HRQL at 3-months (across all domains)
Nicholas et al. [75]	Headache Disorder	THC: CBD	97 (57)	37.9 (11.1)	3-m 6-m [Dec 2019–Feb 2022]	Cohort study	Yes	No	EQ-5D-5L [yes]	Improved HRQL at 3- and 6-months (index score)
Palmieri et al. [87]	Any condition	THC	20 (60)	40.7 (nr)	3-m [nr]	Cohort study	No	No	SF-36 [yes]	Improved HRQL at 3-months (across 6 domains) No change in role limitations and emotional state domains
Peterson et al. [38]	Chronic Pain	THC: CBD	181 (47)	41.21 (12.9)	6-w [‘*Summer and Autumn*’2020]	Cohort study	Yes	Yes [Yes]	EQ-5D-5L [yes]	Improved HRQL at 6-weeks (index score) Worsening in self-care domain
^#^Rifkin-Zybutz et al. [57]	Anxiety disorder	THC: CBD	202 (68.6)	37 (11.5)	3-m 6-m [Dec 2019–nr]	Cohort study	Yes	No	EQ-5D-5L [yes]	Improved HRQL at 3- and 6-months (index score)
Russo et al. [45]	Multiple Sclerosis (Neuropathic Pain)	THC: CBD	20 (40)	46 (5.8)	1-month [nr]	Cohort study	No	No	MSQoL-54 [no]	Improved HRQL at 1-month (physical and mental composite summary scores)
Russo et al. [44]	Multiple Sclerosis spasticity	THC: CBD	61 (nr)	42 (8.9)	1-m 6-m [Jan 2014–Dec 2014]	Cohort study	No	No	MSQoL-54 [yes]	Deterioration of HRQL at 1-month (physical composite summary score) Improved HRQL at 6-months (mental composite summary scores)
Safakish et al. [37]	Chronic Pain	THC: CBD	751 (43)	49.6 (14.3)	3-m 6-m 12-m [Oct 2015–Mar 2019]	Cohort study	Yes	No	SF-12 [yes]	Improved HRQL over 12-months (physical and mental composite summary scores)
Schloss et al. [53]	Cancer (Glioma)	THC: CBD	83 (51)	53.3 (12.6)	12-w [Nov 2018–Dec 2019]	RCT - parallel	No	No	FACT-Br [yes]	No change in HRQL at 12-weeks (total score) Improved sleep and physical wellbeing domains
Sridharan et al. [65]	Fibromyalgia	THC: CBD	148 (19.6)	47.2 (13.5)	3-m 6-m 12-m [Dec 2019–Jan 2023]	Cohort study	Yes	No	EQ-5D-5L [yes]	Improved HRQL at 3-, 6- and 12-months (index score)
Stienrut et al. [84]	Any condition	THC: CBD	21,284 (52.5)	54.1 (15.3)	3-m [Sep 2019–Oct 2020]	Cohort study	Yes	No	EQ-5D-5L [yes]	Improved HRQL at 3-months (index score).
Sultan et al. 2024 [74]	Post-Traumatic Stress Disorder	THC	58 (66)	39.2 (8.9)	3-m 6-m [Aug 2020–Apr 2023]	Cohort study	No	No	EQ-5D-5L [yes]	Improved HRQL at 3- and 6-months (EQ-5D VAS)
^‡^Tait et al. [35]	Chronic Pain	THC: CBD	761 (47.3)	46.8 (15.4)	3-m 6-m [Dec 2019–Jan 2022]	Cohort study	Yes	No	EQ-5D-5L [yes]	Improved HRQL at 3- and 6-months (index score) No improvements at 6 months using MC flower
*Tait et al. [85]	Any condition	CBD/ THC: CBD	2327 (37.2)	51 (15.4)	3-m [Nov 2020–Dec 2021]	Cohort study	Yes	Yes [Yes]	EQ-5D-5L QLQ-C30 [yes]	Improved HRQL at 3- months (EQ-5D index score and QLQ-C30 summary score)
*Tait et al. 2025 [80]	Any condition	CBD/ THC: CBD	2353 (37.2)	50.4 (15.4)	9-m 12-m [Nov 2020–Mar 2023]	Cohort study	Yes	Yes [Yes]	EQ-5D-5L QLQ-C30 [no]	Improved HRQL at 9- and 12-months (EQ-5D index score and QLQ-C30 summary score)
Ueberall et al. [31]	Chronic Pain	THC: CBD	800 (43)	46.3 (9.7)	12-w [Mar 2017–Dec 2017]	Case series	Yes	No	SF-12 [yes]	Improved HRQL at 12-weeks (physical and mental composite summary scores) Deterioration only in patients with nociceptive pain
Varadpande et al. [49]	Cancer Pain	THC: CBD	168 (60.7)	54.2 (14.6)	3-m 6-m [Dec 2019–Dec 2023]	Case series	No	No	EQ-5D-5L [yes]	Improved HRQL at 3- and 6-months (index score)
Vermersch et al. [43]	Multiple Sclerosis spasticity	THC: CBD	433 (44.8)	50.4 (10.4)	3-m [Aug 2013–Oct 2015]	Cohort study	No	No	EQ-5D-5L [no]	Improved HRQL at 3-months (EQ-5D VAS)
Vickery et al. [97]	Any condition	CBD/THC/ THC: CBD	3961 (46)	56.1 (19.2)	3-m 24-m [Dec 2018–Apr 2022]	Cohort study	No	No	SF-36 [yes]	Improved HRQL at 3-, 12-, and 24- months (physical and mental composite summary scores)
Vigano et al., 2023 [86]	Any condition	THC: CBD	2991 (49.8)	50.9 (18–96)	3-m 9-m 12-m [May 2015–Oct 2018]	Cohort study	No	No	EQ-5D-5L [yes]	Improved HRQL at 3- and 12-months (global QoL)
Vivek et al. [70]	Insomnia	THC: CBD	61 (72)	41.3 (13.0)	3-m 6-m [Dec 2019–Feb 2022]	Case series	Yes	No	EQ-5D-5L [yes]	Improved HRQL at 3- and 6-months (index score)
Ware et al. [36]	Chronic Pain	THC: CBD	431 (51.6)	45.5 (19–82)	6-m 12-m [Jan 2004–Apr 2008]	Cohort study	No	No	SF-36 [no]	Improved HRQL compared with control group at 6- and 12-months (physical composite summary score). No change in mental composite summary score
^#^Warner-Levy et al. [56]	Generalised Anxiety Disorder	THC: CBD	302 (69.5)	38.06 (11.7)	3-m 6-m 12-m [Dec 2019–Jan 2023]	Cohort study	Yes	No	EQ-5D-5L [yes]	Improved HRQL at 3-, 6-, and 12-months (index score)

^*^Papers report HRQL from same study cohort (2353 patients in QUEST study) but at different follow-up assessment timepoints (*n* = 2)

^#^Papers report HRQL from same study cohort (302 patients with anxiety on UK Medical cannabis Registry) at same follow-up assessment timepoints (*n* = 4)

^‡^Papers report HRQL from same study cohort (1254 patients with chronic pain on UK Medical cannabis Registry) at overlapping follow-up assessment timepoints (*n* = 7)

^†^Papers report HRQL from same study cohort (312 patients with any condition on UK Medical cannabis Registry) at overlapping follow-up assessment timepoints (*n* = 2)

CBD, cannabidiol (only); CBDV, cannabidivarin; EQ-5D-3 L, EuroQol quality of life − 5 dimensions-3 levels; EQ-5D-5L, EuroQol quality of life − 5 dimensions-5 levels; ESAS, Edmonton Symptom Assessment Score; FACT-Br, Functional Assessment of Cancer Therapy – Brain; FIQ, Fibromyalgia Impact Questionnaire; HRQL, health-related quality of life; IBDQ, Inflammatory bowel disease questionnaire; m, months; MC, medicinal cannabis; MFHW, Marburg questionnaire on habitual health; MSQoL-54, Multiple Sclerosis Quality of Life-54 items; nr, not reported; PDI, Pain Disability Index; PEG, 3-item scale assessing Pain Intensity and Interference; PHQ-4, Patient Health Questionnaire-4 items; PROM, patient-reported outcome measure; PSQI, Pittsburgh Sleep Quality Index; QLQ-C30, Quality of Life Questionnaire - Cancer 30 items; QLQ-C15, Quality of Life Questionnaire - Cancer 15 items for use in palliative care; QoL, quality of life; QOLIE-89, Quality Of Life In Epilepsy-89 items; QOLIE-31-P, Quality Of Life In Epilepsy-31items-plus 8 distress-related items; QOLIE-10, Quality Of Life In Epilepsy-10 items; RCT, randomised controlled trial; ref, reference; SD, standard deviation; SF-36, Short Form Health Survey − 36 items; SF-12, Short Form Health Survey − 12 items; S-TOPS, Treatment Outcomes in Pain Survey-Short Form; THC, delta-9-tetrahydrocannabinol (only); THC: CBD, combination of THC and CBD; VAS, visual analogue Scale w, weeks; WHOQOL-BREF, World Health Organisation Quality of Life Abbreviated Assessment

### Quality and completeness of PRO reporting

**Fig. 3:**
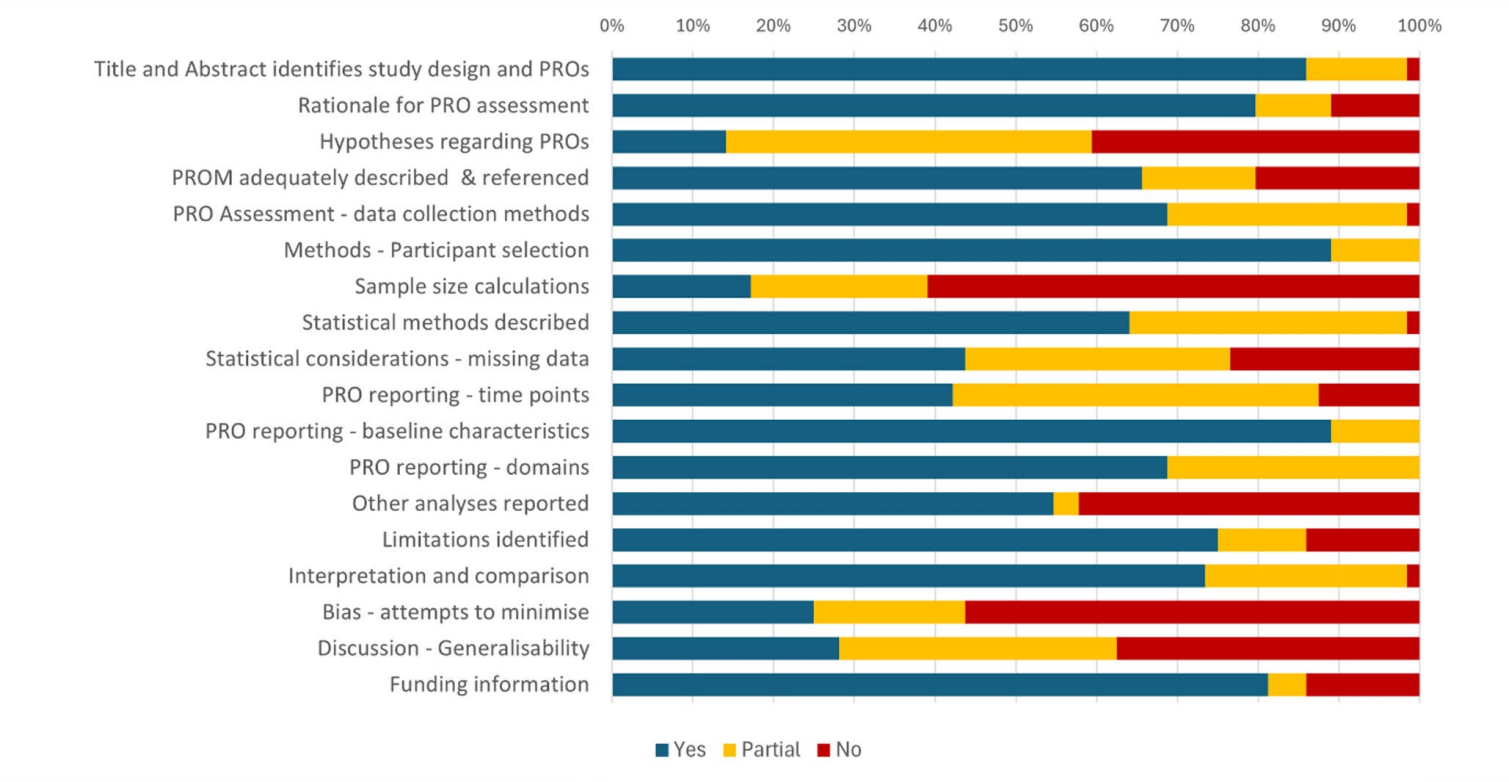
Completeness of PRO reporting for included studies (*n* = 64) displayed as % of criteria met

When evaluating all 64 included studies for completeness of reporting using the composite STROBE and CONSORT-PRO criteria, 40/64 (63%) studies met 75% or more of PRO reporting criteria either fully or partially, and one study failed to meet more than 50% of criteria (Online Resource 5). Figure 3 displays the degree to which each PRO reporting criterion was met across all included studies. All studies reported recruitment procedures, baseline characteristics, and described PRO or HRQL domains. More than half of studies failed to describe sample size calculations or attempts to minimise bias. Looking specifically at the 52 observational studies, 32 (62%) reported no attempts to address bias (i.e., have high risk of bias).

**Fig. 4:**
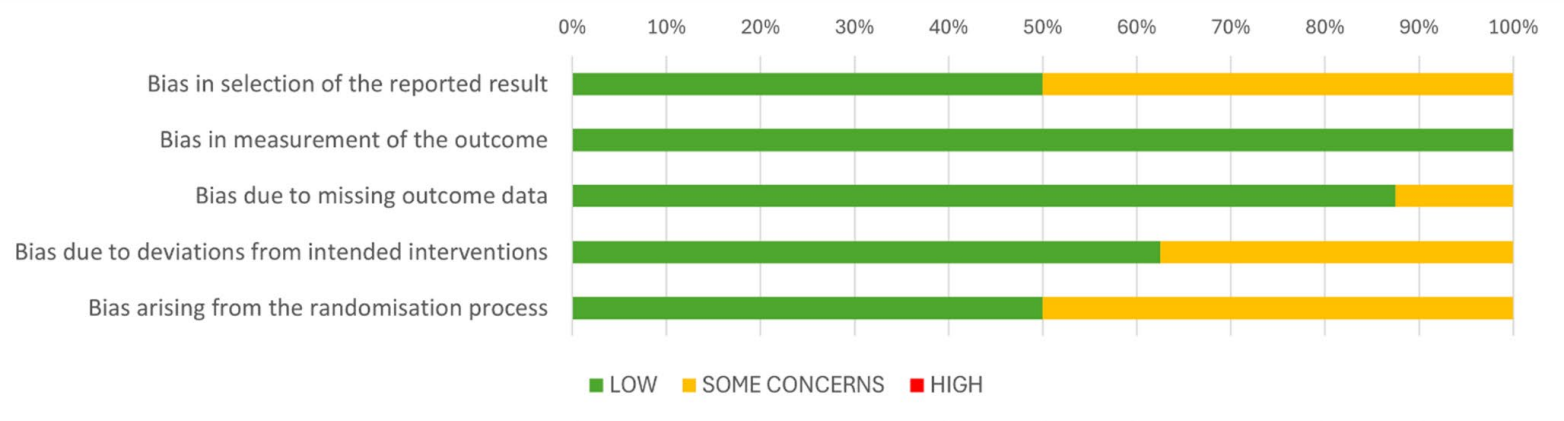
Risk of Bias graph: review of ratings for each RoB 2 criteria presented as percentage across 8 RCTs included in meta-analysis

**Fig. 5:**
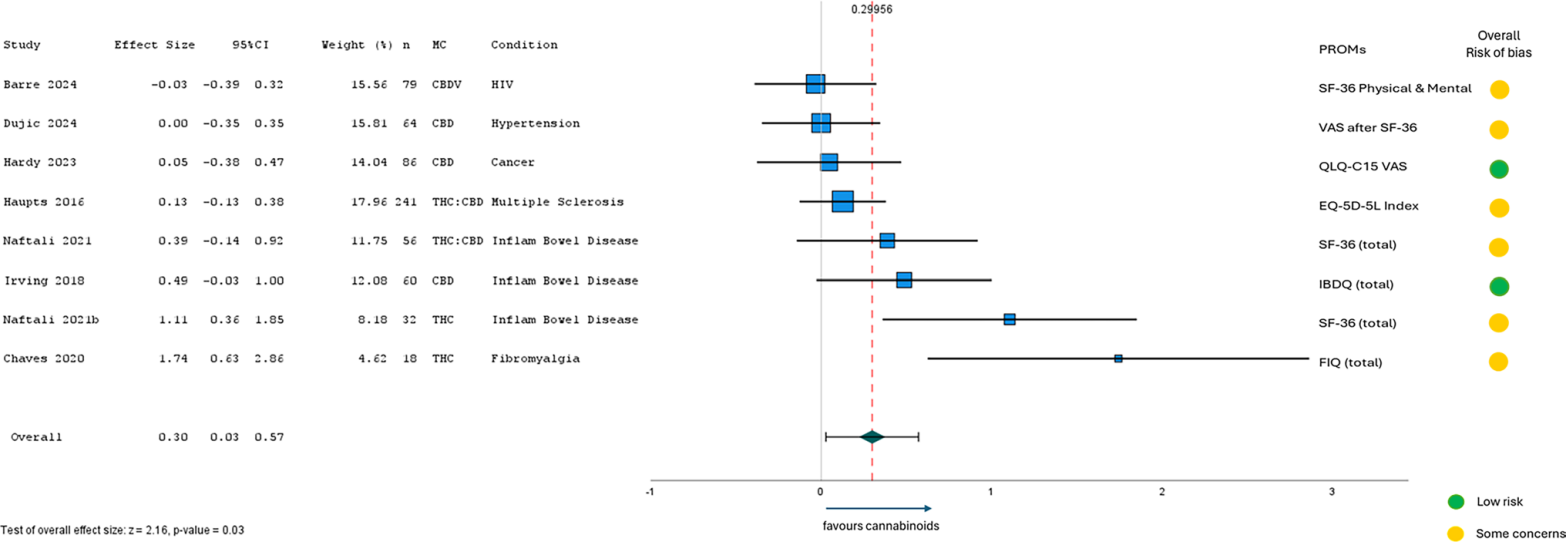
Meta-Analysis Results from RCTs (*n* = 9) assessing short-term HRQL after MC (2-weeks to 3-months)

CBD, cannabidiol; CBDV, cannabidivarin; EQ-5D-5L, EuroQol quality of life − 5 dimensions-5 levels; FIQ, Fibromyalgia Impact Questionnaire; HRQL, health-related quality of life; IBDQ, Inflammatory bowel disease questionnaire; PROM, patient-reported outcome measure; QLQ-C15, Quality of Life Questionnaire - Cancer 15 items for use in palliative care; RCT, randomised controlled trial; SF-36, Short Form Health Survey − 36 items; THC, delta-9 tetrahydrocannabinol; VAS, visual analogue Scale.

None of the 12 RCTs showed high risk of bias on any of the RoB 2 tool criteria and all were evaluated as having low risk of bias in measurement of the outcome (Fig. 4). Ratings for each RCT against RoB 2 items are presented in Online Resource 6, and overall RoB rating added to Fig. 5.

### Meta-analyses by study design

#### Short-term between group differences

Of the 12 studies comparing between group differences (cannabinoid treatment group vs. placebo or controls), eight reported sufficient data to be included in a meta-analysis (Fig. 5). Of excluded studies, one did not report any measure of variability (standard deviations, IQR, or confidence intervals), one reported improvement from baseline but provided post-treatment HRQL scores only, one simply stated the difference was not significant, and one study reported > 30% improvement responder rates across multiple domains. The eight included studies were all RCTs, either parallel (*n* = 7) or crossover (*n* = 1), with treatment periods ranging from 4-weeks to 3-months (short-term). Between group effect sizes ranged from − 0.03 to 1.74, with an overall effect size of 0.30 (95% CI: 0.03 to 0.57; *p* = 0.03; *τ*^2^ = 0.09) favouring cannabinoids. Only two studies found statistically significant HRQL improvements. Across conditions, subgroup analyses by MC composition revealed THC interventions (d = 1.30; 95%CI: 0.68 to 1.92; *p* < 0.001) were more effective for improving HRQL than CBD alone (d = 0.07; 95%CI: -0.13 to 0.27; *p* = 0.47). Meta-analysis was conducted after removing the outlier with effect size greater than ± 1.5 (i.e., Chaves 2020), revealing a small overall effect size of 0.15 (95%CI: 0.01 to 0.30; *p* = 0.04; *τ*^2^ = 0.00). Further subgroup analyses by health condition found MC least effective for improving HRQL in HIV-associated neuropathic pain and hypertension, with larger HRQL improvements in studies of fibromyalgia and Inflammatory Bowel Disease (d = 0.57;95%CI: 0.24 to 0.90; *p* < 0.001).

#### Short-term within group differences

**Fig. 6:**
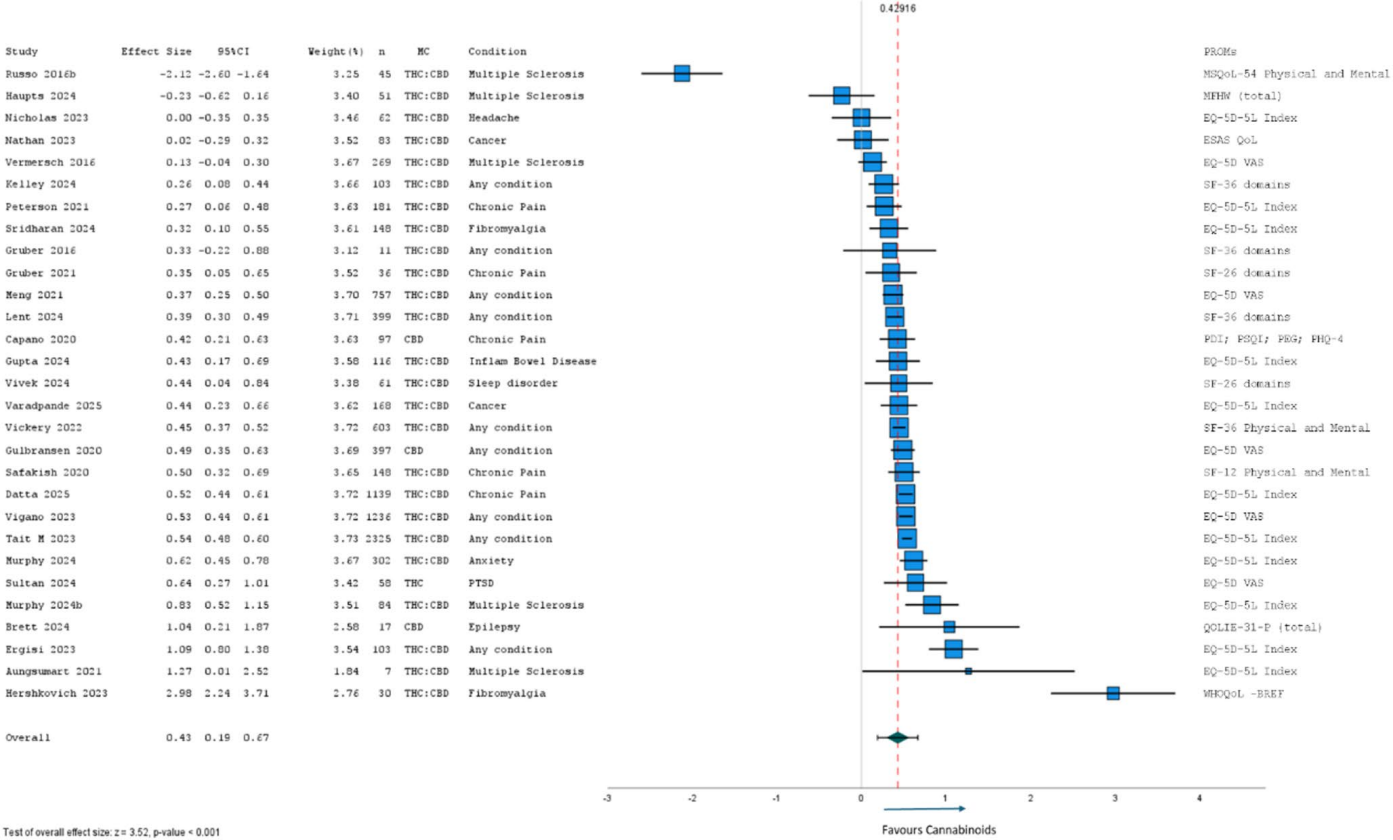
Meta-analysis of short-term (3-weeks to 3-months) results from cohort and case series studies (*n* = 29) of HRQL after MC

Of the 46 studies evaluating short-term within group differences (pre- and post-cannabinoid treatment), 29 reported sufficient data and were eligible for meta-analysis (Fig. 6). Of the 17 excluded studies, 10 assessed participants from an already included study, and seven did not report a measure of variability or usable test statistic. The 29 included studies were all observational, either cohort (*n* = 24) or case series (*n* = 5), with treatment periods ranging from 3-weeks to 3-months (short-term). 79% (*n* = 23) of studies found significant improvements in HRQL over the first 3-months of MC therapy. Within group effect sizes ranged from − 2.12 to 2.98, with an overall effect size of 0.43 (95% CI: 0.19 to 0.67; *p* < 0.001; *τ*^2^ = 0.40) favouring cannabinoids. As with RCTs, meta-analysis was also conducted after removing outliers with effect sizes greater than ± 1.5 (i.e., Russo 2016b, and Hershkovich 2023), maintaining an overall moderate effect size of 0.43 (95%CI 0.34 to 0.51; *p* < 0.001; *τ*^2^ = 0.03). Further subgroup analyses revealed similar HRQL improvements in studies investigating CBD only (d = 0.48; 95%CI: 0.36 to 0.60; *p* < 0.001) and those investigating various THC: CBD combinations (d = 0.41; 95%CI: 0.31 to 0.51; *p* < 0.001). Analyses by health condition revealed largest HRQL improvements for PTSD, anxiety, chronic pain (d = 0.45; 95%CI: 0.35 to 0.55; *p* < 0.001), and studies across any health condition (d = 0.48; 95%CI: 0.37 to 0.60; *p* < 0.001). Studies investigating headache, cancer (d = 0.24; 95%CI: -1.74 to 0.66; *p* = 0.252), and multiple sclerosis (d = 0.38; 95%CI: -0.21 to 0.96; *p* = 0.204) found no significant changes in HRQL. Short-term HRQL changes from baseline were most variable in studies of patients with multiple sclerosis, where an equal number of studies reported improved HRQL or no change.

CBD, cannabidiol; EQ-5D-5L, EuroQol quality of life − 5 dimensions-5 levels; HRQL, health-related quality of life; MC, medicinal cannabis; MFHW, Marburg questionnaire on habitual health; MSQoL-54, Multiple Sclerosis Quality of Life-54 items; PDI, Pain Disability Index; PEG, 3-item scale assessing Pain Intensity and Interference; PHQ-4, Patient Health Questionnaire-4 items; PROM, patient-reported outcome measure; PSQI, Pittsburgh Sleep Quality Index; QOLIE-31-P, Quality Of Life In Epilepsy-31items-plus 8 distress-related items; SF-36, Short Form Health Survey − 36 items; SF-12, Short Form Health Survey − 12 items; THC, delta-9 tetrahydrocannabinol; VAS, visual analogue Scale; WHOQOL-BREF, World Health Organisation Quality of Life Abbreviated Assessment.

#### Medium-term within group differences

**Fig. 7:**
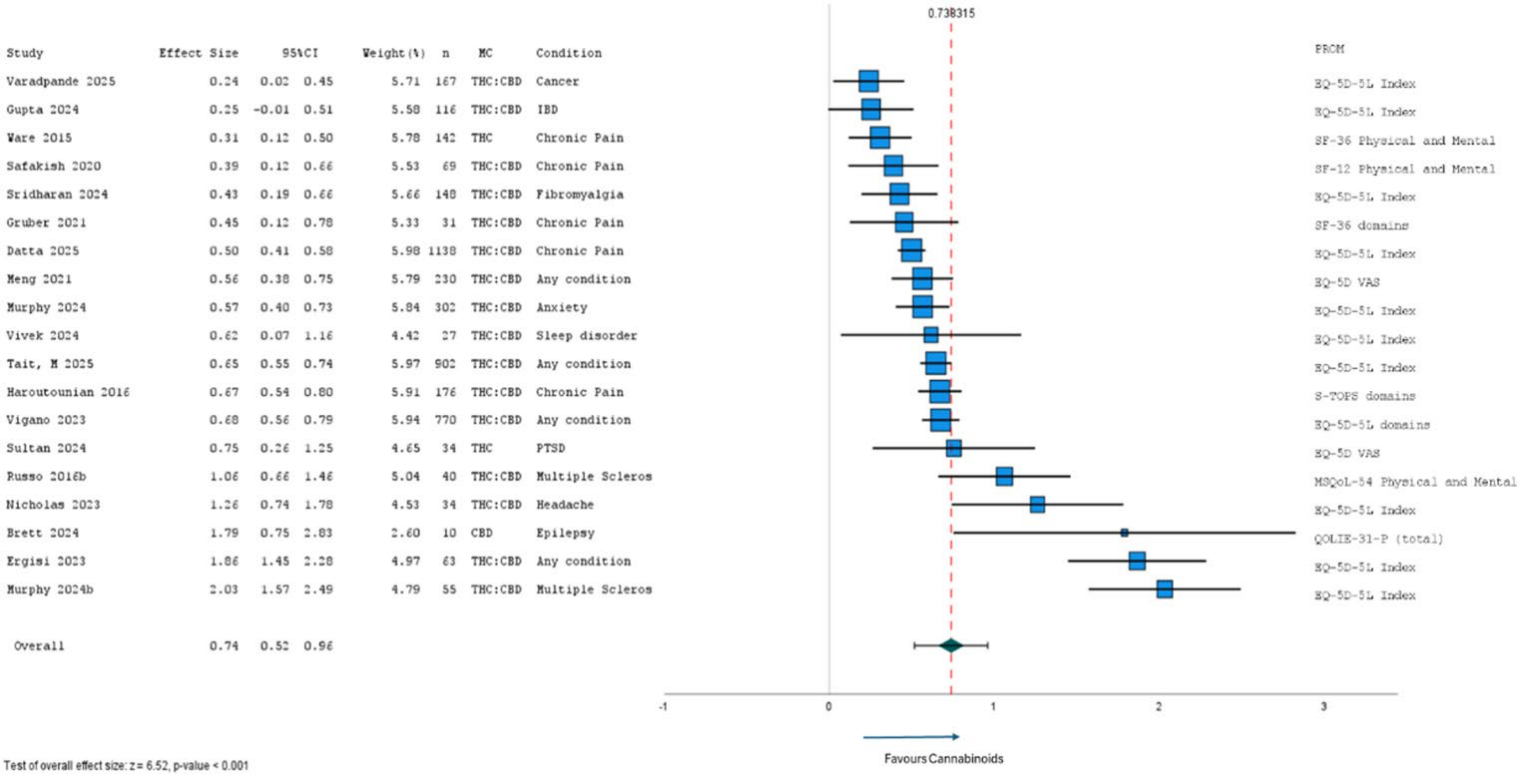
Meta-analysis of medium-term (5–9 months) results from cohort and case series studies (*n* = 19) of HRQL after MC

CBD, cannabidiol; EQ-5D-5L, EuroQol quality of life − 5 dimensions-5 levels; HRQL, health-related quality of life; IBD, Inflammatory Bowel Disease; MC, medicinal cannabis; MSQoL-54, Multiple Sclerosis Quality of Life-54 items; PROM, patient-reported outcome measure; QOLIE-31-P, Quality Of Life In Epilepsy-31items-plus 8 distress-related items; SF-36, Short Form Health Survey − 36 items; SF-12, Short Form Health Survey − 12 items; S-TOPS, Treatment Outcomes in Pain Survey-Short Form; THC, delta-9 tetrahydrocannabinol; VAS, visual analogue Scale.

Of the 30 studies evaluating medium-term within group differences (pre- and post-cannabinoid treatment), 19 were included in meta-analysis (Fig. 7). Of the 11 excluded studies, nine assessed participants from an already included study, and two did not report a measure of variability or usable test statistic. The 19 included studies were all observational, either cohort (*n* = 14) or case series (*n* = 5), with treatment assessment periods of 5-months (*n* = 1), 6-months (*n* = 16), or 9-months (*n* = 2). 95% (*n* = 18) of studies reported significantly improved HRQL. Within group effect sizes ranged from 0.24 to 2.03, with an overall effect size of 0.74 (95% CI: 0.52 to 0.96; *p* < 0.001; *τ*^2^ = 0.21) favouring cannabinoids. A few outliers with effect sizes greater than 1.5 influenced overall effect size. Meta-analysis was conducted after removing outliers with effect sizes greater than ± 1.5 (i.e., Brett 2024, Murphy 2024b, and Ergasi 2023), revealing an overall moderate effect size of 0.54 (95%CI: 0.45 to 0.64; *p* < 0.001; *τ*^2^ = 0.02). Further subgroup analyses revealed greater HRQL improvement over the medium term in studies investigating various THC: CBD combinations (d = 0.56; 95%CI: 0.46 to 0.66; *p* < 0.001) compared with THC only (d = 0.47; 95%CI: 0.05 to 0.89; *p* = 0.029). Larger HRQL improvements were seen across studies assessing patients with headache, multiple sclerosis, PTSD, chronic pain (d = 0.48; 95%CI: 0.35 to 0.62; *p* < 0.001) and any condition (d = 0.64; 95%CI: 0.58 to 0.71; *p* < 0.001).

#### Long-term within group differences

**Fig. 8:**
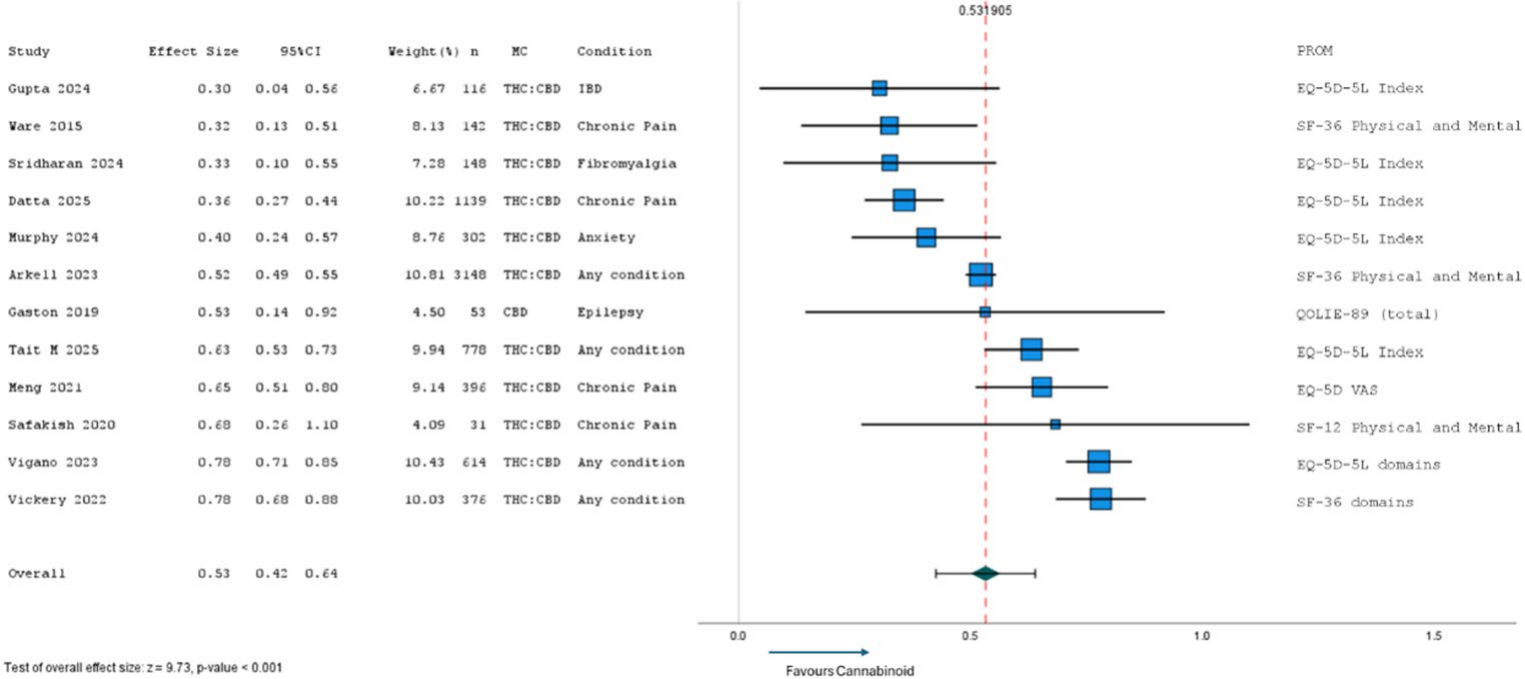
Meta-analysis of long-term (12–24 months) HRQL results from cohort and case series studies (*n* = 12) after MC therapy. CBD, cannabidiol; EQ-5D-5L, EuroQol quality of life − 5 dimensions-5 levels; HRQL, health-related quality of life; IBD, Inflammatory Bowel Disease; MC, medicinal cannabis; PROM, patient-reported outcome measure; QOLIE-89, Quality Of Life In Epilepsy-89 items; SF-36, Short Form Health Survey − 36 items; SF-12, Short Form Health Survey − 12 items; THC, delta-9 tetrahydrocannabinol; VAS, visual analogue Scale

Of the 17 studies evaluating long-term within group differences (pre- and post-cannabinoid treatment), five were excluded because they assessed participants from an already included study, leaving 12 studies with sufficient data to include in meta-analysis (Fig. 8). Included studies were all observational, either cohort (*n* = 9) or case series (*n* = 3), with treatment assessment periods of 12-months (*n* = 9), 18-months (*n* = 1), 20.7-months (*n* = 1), or 24-months (*n* = 1). Within group effect sizes ranged from 0.30 to 0.78, with an overall effect size of 0.53 (95% CI: 0.42 to 0.64; *p* < 0.001; *τ*^2^ = 0.03) favouring cannabinoids. All studies found significant improvement in HRQL from 12-months. Further subgroup analyses found the largest HRQL improvements across studies investigating any health condition (d = 0.67; 95%CI: 0.55 to 0.80; *p* < 0.001), chronic pain (d = 0.47; 95%CI: 0.29 to 0.66; *p* < 0.001), and anxiety.

## Discussion

This systematic review with meta-analysis included all prospective studies between 2015 and 2025 reporting HRQL in patients treated with non-synthetic MC. The included studies are mostly representative of countries globally where MC is legal, apart from southeastern African countries where MC is legal, but no studies were identified.

Completeness of PRO reporting was adequate for most studies, however one study failed to meet more than 50% of reporting criteria. HRQL was rarely defined and a third of studies did not provide a rationale for assessing HRQL. Failing to define HRQL, justify its assessment, or provide a clear HRQL hypothesis suggests these studies may not have had a clear research objective regarding HRQL. PROs are increasingly being used in clinical studies [98], following recommendations from professional and clinical organisations, health agencies, and regulatory bodies [10, 99, 100]. It is important for researchers to be explicit about the meaning and context of HRQL in their studies to enable assessment of whether the chosen HRQL measure was fit-for-purpose [14]. We found vastly different short-term HRQL results across the six observational studies assessing patients with multiple sclerosis treated with THC: CBD. Although not directly assessed, this may partly be due to a lack of HRQL definitions and rationales for evaluating HRQL, and the use of different HRQL PROMs across these studies. Having a clear HRQL definition helps inform the selection of appropriate PROMs, improves scientific rigor, and helps contextualise any discrepancies in findings. This is particularly important considering different PROMs have previously been shown to produce discrepant HRQL results within the same group of patients [14].

Overall, our meta-analysis findings suggest that, compared with baseline, small to moderate HRQL improvements are apparent within 3 months of commencing MC, with moderate to large improvements between 5 and 9-months, and moderate levels long-term (12 to 24 months). However, HRQL did not improve for all conditions and improvements often varied depending on cannabinoids used. Findings across moderate- to high-quality RCTs indicated clinically meaningful short-term improvements in HRQL were mostly driven by studies of patients with irritable bowel disease or fibromyalgia. One study with HIV patients used a specific cannabinoid, Cannabidivarin (CBDV), not investigated by any other included studies, which focused on CBD and THC cannabinoids. The findings from this study suggest CBDV was not effective in this cohort for reducing pain or improving HRQL. Meta-analysis of short-term observational studies found moderate HRQL improvements overall, with similar findings across studies looking at CBD only and THC: CBD in combination. However, this was also dependent on health conditions treated, with no change seen across studies of patients with cancer or multiple sclerosis. Notably, the two observational studies providing extreme negative and positive effect sizes were also rated as having low completeness of PRO reporting. For example, Hershkovich et al. didn’t specify the type of variability reported in results, however, after due diligence and investigation, we determined it was most likely standard deviation and included it in the meta-analysis. Although doubts were raised about the accuracy of reporting (e.g. whether standard error was mistakenly reported as standard deviation), we assumed research findings in peer-reviewed publications were reported correctly. However, to reduce possible bias from erroneous data, we crossed-checked all statistical information available for determining study effect sizes, including t tests and reported p-values, and where relevant, chose the most conservative estimation for inclusion in meta-analyses.

### Comparisons with previous review findings

In their review, Goldenberg et al. found no main effect of MC on HRQL [15]. Along with synthetic cannabinoids, they reviewed six studies that used cannabis (smoked or ingested) and seven that used non-synthetic pharmaceutical cannabinoids (Sativex). Regarding the six cannabis studies, they found no main effect on HRQL, however, three of the studies were cross-sectional without pre- and post-therapy comparisons, two were prospective with small sample sizes (13 and 37), with the one RCT reporting significant but small improvements in mental HRQL in patients with neuropathic pain [101]. Across the seven non-synthetic cannabinoid studies, six found no main effect of MC on HRQL, and one observational study of 276 multiple sclerosis patients found significant HRQL improvements when assessed with MSQoL-54, but no HRQL change when the same patients were assessed using EQ-5D-3 L [102]. We observed similar disparate HRQL findings across studies assessing HRQL in multiple sclerosis patients using different PROMs, further highlighting the need to clearly define HRQL in this context to inform PROM selection. Goldenberg et al. concluded that where HRQL improvements were reported, this was mainly attributed to pain reduction [15]. However, as summarised in Table 1, we found that studies reported HRQL improvements in patients due not only to pain reduction, but also through resolving sleep, and physical and psychological issues [41, 42, 48, 53–57, 62–64, 66, 70, 71, 79].

## Strengths and limitations

A strength of this review is that all studies reviewed used non-synthetic cannabinoids derived from the cannabis plant, reflective of current MC products available in countries where MC prescribing is legal. Further, studies followed participants across short-, medium-, and long-term follow-up with meta-analyses comparing studies with similar follow-up assessment periods. A further strength of the current review is the wide coverage of 77 health conditions treated with MC in included studies (Online resource 4). This contrasts with previous reviews of HRQL in MC that included studies of synthetic cannabinoids, had limited coverage of health conditions, and short follow-up periods [15, 16].

Generally, meta-analysis of observational studies should be interpreted cautiously, particularly over medium- and long-term assessment periods, due to possible attrition bias [103]. This is a limitation of review findings because participants remaining in observational studies are more likely those who are benefitting from therapy, inflating positive findings. Across all studies included in this review, a quarter employed methods to reduce attrition bias including intention-to-treat analyses in RCTs [6, 31, 47, 48, 53, 61], and applying the ‘baseline observation carried forward approach’ [104] for handling missing data in observational studies [30, 33, 49, 54–56, 58, 65, 68, 69]. However, when reasons for missing data are unknown, imputing data may potentially introduce other biases. We found most included observational studies did not report any attempts to reduce or address bias in their studies.

Another limitation of interpreting results of within group meta-analysis is that the correlation between pre and post therapy scores was often not reported in studies, thus introducing biased effect size variance, and without a control group it is unknown whether the effect is due to therapy, placebo, or natural recovery [105]. To account for this, we applied recommended correlations where available for repeated measures (including for RCT-crossover trials) and for multiple outcomes (HRQL domains) reported by the same group [28, 29]. This is in contrast to a previous meta-analysis that included multiple domains from one study alongside studies with single outcomes, inflating the overall effect size contributions from studies with multiple domains [15].

## Conclusions

We found a main effect of improved HRQL in patients using MC across studies published over the past decade. Improvements were observed across multiple health conditions over short-, medium- and long-term follow-up. However, disparate findings between studies of patients with the same health condition were also observed, possibly due to using different HRQL measures. This conflicting evidence may be better understood when researchers communicate how HRQL is defined and assessed in the context of their study and provide a justification for PROM selection.

## Supplementary Information

Below is the link to the electronic supplementary material.

Supplementary Material 1

Supplementary Material 2

Supplementary Material 3

Supplementary Material 4

Supplementary Material 5

Supplementary Material 6

## Data Availability

No datasets were generated or analysed during the current study.
