# Correction: Health-related quality of life in patients receiving medicinal cannabis: systematic review and meta-analysis of primary research findings 2015–2025

**DOI:** 10.1007/s11136-026-04216-w

**Published:** 2026-03-25

**Authors:** Margaret-Ann Tait, Louise Acret, Daniel S. J. Costa, Rachel Campbell, Kate White, Claudia Rutherford

**Affiliations:** 1 Faculty of Medicine and Health, Susan Wakil School of Nursing, The University of Sydney, Sydney, NSW Australia; 2 Sydney Local Health District, Sydney, NSW Australia; 3 The Daffodil Centre, The University of Sydney, and Cancer Council NSW, Sydney, NSW Australia; 4 Faculty of Science, School of Psychology, The University of Sydney, Sydney, NSW Australia

**Correction to: Quality of Life Research (2026) 35:56**10.1007/s11136-026-04170-7.

In this article, Fig. 5 appeared incorrectly. For completeness and transparency, the old incorrect and correct versions are displayed in this correction.

Incorrect figure:

**Fig. 5:**
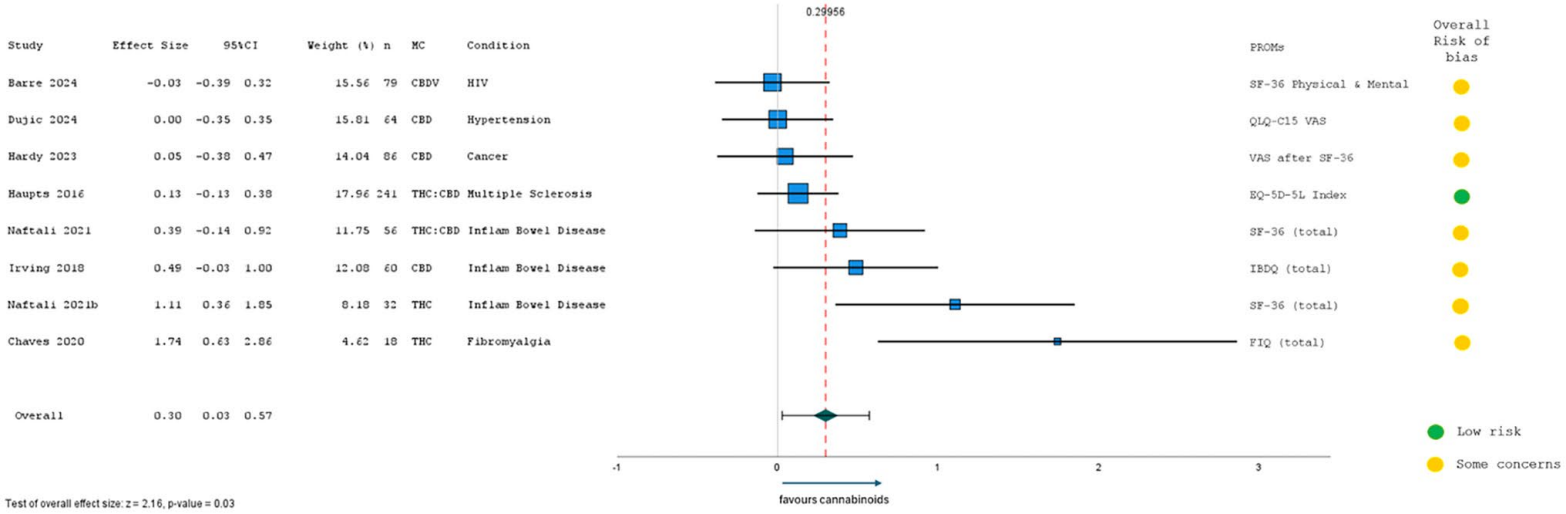
Meta-Analysis Results from RCTs (n = 9) assessing short-term HRQL after MC (2-weeks to 3-months)

Correct figure:

**Fig. 5:**
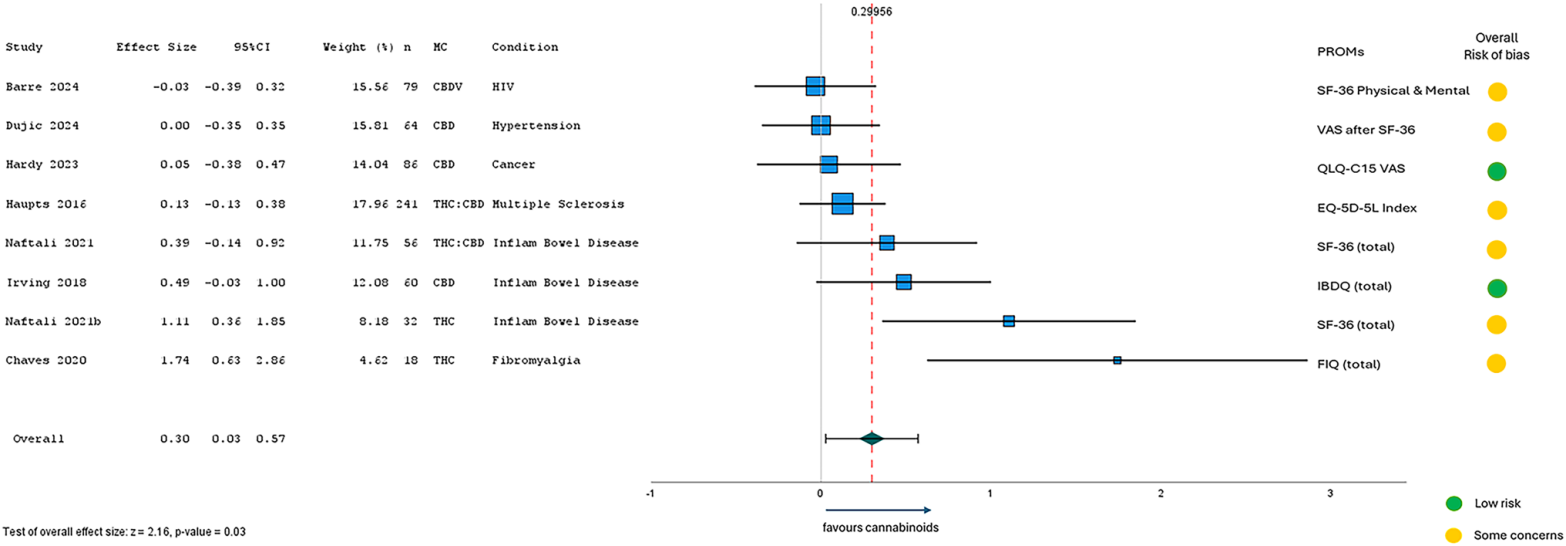
Meta-Analysis Results from RCTs (n = 9) assessing short-term HRQL after MC (2-weeks to 3-months)

The original article has been corrected.

