# Supplementary material for: Health-related quality of life in patients receiving medicinal cannabis: systematic review and meta-analysis of primary research findings 2015–2025
Source: Qual Life Res. 2026 Feb 1;35(3):56. doi: 10.1007/s11136-026-04170-7 (PMC12862010; doi:10.1007/s11136-026-04170-7)
Supplement: Supplementary file 1 — Supplementary Material 1 [file 11136_2026_4170_MOESM1_ESM.pdf]

Health-related quality of life in patients receiving medicinal cannabis: Systematic review and meta-analysis of primary research findings 2015 – 2025

Quality of Life Research

*Margaret-Ann Tait,[1,2,3], Louise Acret,[1,2,3] Daniel SJ Costa,[4] Kate White,[1,2,3] Rachel Campbell,[4] Claudia Rutherford[1,2,3]
[1]Susan Wakil School of Nursing, Faculty of Medicine and Health, University of Sydney, NSW, Australia
[2]Sydney Local Health District, NSW, Australia
[3]The Daffodil Centre, The University of Sydney, and Cancer Council NSW
[4]School of Psychology, Faculty of Science, University of Sydney, NSW, Australia
* margaret-ann.tait@sydney.edu.au

Online Resource 1: MEDLINE (Ovid) search strategy

1.	Cannabis/
2.	Medical Marijuana/
3.	exp Cannabinoids/
4.	cannabi*.mp. [mp=title, abstract, original title, name of substance word, subject heading word, floating sub-heading word, keyword heading word, organism supplementary concept word, protocol supplementary concept word, rare disease supplementary concept word, unique identifier, synonyms]
5.	marijuana.mp.
6.	CBD.mp.
7.	Tetrahydrocannabinol.mp.
8.	THC.mp.
9.	sativ*.mp.
10.	Phytotherapy/
11.	nabiximol.mp.
12.	hemp.mp.
13.	epidiolex.mp.
14.	phytocannabinoid.mp.
15.	1 OR 2 OR 3 OR 4 OR 5 OR 6 OR 7 OR 8 OR 9 OR 10 OR 11 OR 12 OR 13 OR 14
16.	"Quality of Life"/
17.	"Surveys AND Questionnaires"/ OR patient reported outcome measures.mp. OR Patient Reported Outcome Measures/
18.	Symptom Assessment/ OR symptom*.mp.
19.	questionnaire.mp.
20.	HRQL.mp.
21.	HRQOL.mp.
22.	(health-related adj 'quality of life').mp.
23.	(patient* adj2 interview*).mp.
24.	16 OR 17 OR 18 OR 19 OR 20 OR 21 OR 22 OR 23
25.	15 AND 24
26.	limit 25 to (english language AND yr="2015 -Current")
27.	limit 26 to (humans AND "all adult (19 plus years)")
28.	27 NOT (address OR autobiography OR bibliography OR biography OR clinical conference.pt OR clinical trial, veterinary OR clinical trials, veterinary as topic OR clinical trial protocol OR clinical trial protocols as topic OR comment OR congress OR consensus development conference OR consensus development conference, nih OR dictionary OR directory OR editorial OR english abstract OR "expression of concern" OR historical article OR interactive tutorial OR lecture OR legal case OR legislation OR letter OR news OR newspaper article OR observational study, veterinary OR patient education handout OR periodical index OR portrait OR randomized controlled trial, veterinary OR technical report OR video-audio media OR webcast).pt.
29.	27 not 28
30.	limit 29 to (abstracts AND pharmacologic actions)
31.	29 NOT 30
