# Supplementary material for: Health-related quality of life in patients receiving medicinal cannabis: systematic review and meta-analysis of primary research findings 2015–2025
Source: Qual Life Res. 2026 Feb 1;35(3):56. doi: 10.1007/s11136-026-04170-7 (PMC12862010; doi:10.1007/s11136-026-04170-7)
Supplement: Supplementary file 2 — Supplementary Material 2 [file 11136_2026_4170_MOESM2_ESM.pdf]

Health-related quality of life in patients receiving medicinal cannabis: Systematic review and meta-analysis of primary research findings 2015 – 2025

Quality of Life Research

*Margaret-Ann Tait,[1,2,3], Louise Acret,[1,2,3] Daniel SJ Costa,[4] Kate White,[1,2,3] Rachel Campbell,[4] Claudia Rutherford[1,2,3]
[1]Susan Wakil School of Nursing, Faculty of Medicine and Health, University of Sydney, NSW, Australia
[2]Sydney Local Health District, NSW, Australia
[3]The Daffodil Centre, The University of Sydney, and Cancer Council NSW
[4]School of Psychology, Faculty of Science, University of Sydney, NSW, Australia
* margaret-ann.tait@sydney.edu.au

Online Resource 2: PRO reporting criteria adapted from STROBE and CONSORT-PRO tools.

1. Title and Abstract identifies study design and PROs

Study design indicated with a commonly used term in the title or the abstract (e.g. RCT or observational/prospective) AND the abstract summarises design, methods, results, and conclusions AND PROs are clearly identified in the abstract as a primary or secondary outcome.

2. Rationale for PRO assessment

Did the authors provide a background and rationale for PRO assessment?

Yes: The paper provided the background and rationale for the PRO assessment.

Partial: The paper cited some relevant PRO literature but did not provide a rationale for PRO assessment for this study.

No: The paper did NOT provide the background or rationale for the PRO assessment.

3. Hypotheses regarding PROs

Did the authors state the a priori objectives and /or hypotheses about PROs?

Yes: The PRO hypotheses/objective regarding relevant PRO domain(s) are explicitly stated.

Partial: PRO hypotheses/objective are implied but not explicitly stated.

No: PRO hypotheses/objective are neither implied nor stated.

4. Did the authors adequately justify and describe the PRO measures (PROM(s))?

Yes: Choice of PROM(s) is justified by their coverage of the relevant PRO domains. Evidence of reliability/validity of PROM(s) should be referenced. Scoring of items/questions into domains/scales should be described, along with direction of scores (e.g. higher scores equal better QOL).

Partial: Choice of PROM(s) is not justified; authors simply state which PROM(s) they used with a relevant reference (may or may not explicitly mention reliability/validity). Scoring not mentioned.

No: Choice of PROM(s) is not justified; authors simply state which PROM(s) they used with any references. Scoring not mentioned.

5. PRO Assessment - data collection methods

Did the authors adequately describe PRO data collection methods?

Yes: Authors describe how the PROM was administered, and when completed, e.g. "PROs assessed before treatment, 6-wks post, and 6-mths post. Trained nurse administered all questionnaires in clinic"; "patients completed PROs in clinic before treatment and then 6-wks after at home via online data collection platform".

Partial: Some but not complete description of data collection methods.

No: Data collection methods not reported (i.e. it is not clear how the PRO was administered, by whom, and where or when it was completed).

6. Methods - Participant selection

Are the eligibility criteria, and the sources and methods of selection of participants and their groupings (e.g. random allocations, exposed/unexposed, criteria for matched controls) provided?

7. Sample size

Was sample size/power appropriate to detect PRO differences?

Yes: PRO sample size was justified in terms of a priori effect size (e.g. 0.5 SD) or mean difference, type I error rate (alpha), or power – or sample size calculation given in these terms.

Partial: Power justified post hoc [e.g. only mentioned "we had the power to detect differences".

No: No sample size or power calculations mentioned or <10 participants in each treatment group.

8. Statistical methods

Were all statistical methods, including those used to control for confounding described?

(including adjustments for assessment of multiple PRO domains, e.g. Bonferroni)

9. Statistical considerations - missing data

Were statistical approaches for dealing with missing data explicitly stated?

Yes: The extent of missing data and statistical methods for dealing with missing data were both clearly stated.

Partial: The extent of missing data and statistical methods for dealing with it were vague or only one stated.

No: The study did not mention the extent of missing data or how missing data was handled.

10. PRO reporting - time points

Was a description (e.g. flow diagram) of participants through the study reported in relation to PROs, including information on the reason for missing PRO data?

Yes: PRO completion rates were reported for each time point, and reasons for missed assessments reported

Partial: PRO completion reported for some but not all time points AND/OR reasons missing NOT reported for each time point or are unclear.

No: no description of sample size for each timepoint.

11. PRO reporting - baseline characteristics

Did the authors adequately describe baseline sample characteristics for the PRO sample?

Yes: an informative set of baseline PRO demographic and clinical characteristics is reported for each health indication and medcan therapy received (either in table or text).

Partial: Some baseline PRO demographic and clinical characteristics are reported, or an informative set but not at the level of indications and therapy.

No: No baseline/demographic information provided.

12. PRO reporting - domains

Were PRO results reported adequately for all domains? (e.g. mean and SD, median and IQR, or proportion cases above a cut-point. The estimated effect size and its precision such as 95% confidence interval should be presented for multidimensional PROs from each domain and time point.)

Yes: PRO results were reported for all PRO domains measured including effect size and 95%CI.

Partial: PRO results reported for some but not all domains measured (i.e. selective reporting, e.g. may report only statistically significant domains – prone to reporting bias).

13. Other analyses

Did they report other PRO analyses done—eg analyses of subgroups and interactions, and sensitivity analyses?

14. Limitations

Were PRO specific limitations and implications for generalizability addressed? For example, if PRO assessments are limited to a subgroup of the main study population, reasons why patients were excluded from the PRO study should be provided (such as where appropriate translations were unavailable). If PRO data are missing, it is particularly important to discuss the potential reasons in relation to the clinical context and implications for interpretation, e.g. "Non-completers at one year might have had a different symptom profile and overall quality of life than completers, and therefore some degree [of] selection bias is possible"

> **Yes:** Identified sources of potential bias or imprecision when discussing PRO-specific limitations and implications, including both direction and magnitude of any potential bias.
>
> **Partial:** Identified some PRO limitations and their implications.
>
> **No:** Did not address PRO-specific limitations

15. Interpretation

Was PRO data interpreted in relation to clinical outcomes? Did they give a cautious overall interpretation of results considering objectives, limitations, multiplicity of analyses, results from similar studies, and other relevant evidence?

> **Yes:** Interpretation of PRO results include discussion of a minimal important change or a responder definition (e.g. >30% improvement on validated Pain scale) and clinical significance of PRO results were linked to the other trial outcomes such as adverse events, and comparisons made with other similar studies.
>
> **Partial:** Partial interpretations and comparisons of PRO results.
>
> **No:** Findings not supported by results; or findings clearly due to low power are reported as definitive evidence against the alternate hypothesis or interpreted favourably using statistical significance when findings may not be clinically important.

16. Bias

Did they describe any efforts to address potential sources of bias?

17. Discussion - Generalisability

Did they discuss the generalisability (external validity) of the study results?

18. Other information - Funding

Did they give the source of funding (or state no funding) and the role of the funders for the present study and, if applicable, for the original study on which the present article is based?
