# Supplementary material for: Health-related quality of life in patients receiving medicinal cannabis: systematic review and meta-analysis of primary research findings 2015–2025
Source: Qual Life Res. 2026 Feb 1;35(3):56. doi: 10.1007/s11136-026-04170-7 (PMC12862010; doi:10.1007/s11136-026-04170-7)
Supplement: Supplementary file 3 — Supplementary Material 3 [file 11136_2026_4170_MOESM3_ESM.pdf]

Health-related quality of life in patients receiving medicinal cannabis: Systematic review and meta-analysis of primary research findings 2015 – 2025

Quality of Life Research

*Margaret-Ann Tait,[1,2,3], Louise Acret,[1,2,3] Daniel SJ Costa,[4] Kate White,[1,2,3] Rachel Campbell,[4] Claudia Rutherford[1,2,3]
[1]Susan Wakil School of Nursing, Faculty of Medicine and Health, University of Sydney, NSW, Australia
[2]Sydney Local Health District, NSW, Australia
[3]The Daffodil Centre, The University of Sydney, and Cancer Council NSW
[4]School of Psychology, Faculty of Science, University of Sydney, NSW, Australia
* margaret-ann.tait@sydney.edu.au

Online Resource 3. Overview of 64 included studies (ordered by year of publication)

1st Author, year	Country	Title	Study Aims	Journal
Dickinson et al., et al., 2025	United Kingdom	UK Medical Cannabis Registry: An Analysis of Outcomes of Medical Cannabis Therapy for Hypermobility-Associated Chronic Pain	To evaluate clinical outcomes in patients with hypermobility spectrum disorder and hypermobile Ehlers-Danlos syndrome with chronic pain following MC treatment.	ACR Open Rheumatology
Varadpande et al., et al., 2025	United Kingdom	UK Medical Cannabis Registry: An Analysis of Clinical Outcomes of Medicinal Cannabis Therapy for Cancer Pain	To evaluate the change in PROMs of patients enrolled in the UKMCR prescribed MC for chronic pain management.	Journal of Pain and Palliative Care Pharmacotherapy
Datta et al., et al., 2025	United Kingdom	UK medical cannabis registry: A clinical outcome analysis of medical cannabis therapy in chronic pain patients with and without co-morbid sleep impairment	To assess PROMs following MC treatment in Chronic Pain patients with and without co-morbid sleep impairment.	Pain Practice
Tait M, et al., 2025	Australia	Improvements in health-related quality of life are maintained long-term in patients prescribed medicinal cannabis in Australia: The QUEST Initiative 12-month follow-up observational study.	To assess 12-month follow-up data to determine if our previously reported improvements at 3-months were maintained long-term and to explore differences across health conditions and MC compositions.	PloS one
Francis et al., 2024	United Kingdom	Assessment of Clinical Outcomes in Patients With Osteoarthritis: Analysis from the UK Medical Cannabis Registry	To evaluate pain-specific and general HRQL changes in patients with osteoarthritis using patient-reported outcome measures.	Journal of Pain and Palliative Care Pharmacotherapy
Gupta et al., 2024	United Kingdom	UK medical cannabis registry: an updated analysis of clinical outcomes of cannabis-based medicinal products for inflammatory bowel disease	To evaluate differences in PROMs for patients prescribed MC for IBD over an 18-month period using data collected from the UKMCR.	Expert Review of Gastroenterology and Hepatology
Sultan et al., 2024	United Kingdom	Controlled Inhalation of Tetrahydrocannabinol-Predominant Cannabis Flos Mitigates Severity of Post-Traumatic Stress Disorder Symptoms and Improves Quality of Sleep and General Mood in Cannabis-Experienced UK Civilians: A Real-World, Observational Study	To investigate the safety and effectiveness of THC-predominant cannabis flowers for inhalation to manage PTSD symptoms in a real-world setting.	Medical Cannabis and Cannabinoids
Stienrut et al., 2024	Thailand	Medical Cannabis Prescription Practices and Quality of Life in Thai Patients: A Nationwide Prospective Observational Cohort Study	To explore the prescribing patterns of traditional practitioners and assess the impact of cannabis oil on patients' quality of life, with a specific focus on comparing outcomes between cancer and non-cancer patients.	Medical Cannabis and Cannabinoids

1st Author, year	Country	Title	Study Aims	Journal
Warner-Levy et al., 2024	United Kingdom	UK Medical Cannabis Registry: a cohort study of patients prescribed cannabis-based oils and dried flower for generalised anxiety disorder	To investigate real-world outcomes and safety of different MC formulations in GAD patients.	Expert Review of Neurotherapeutics
Lamonarca et al., 2024	Argentina	Psychiatric comorbidities before and after cannabidiol treatment in adult patients with drug resistant focal epilepsy	To evaluate, in the same patient population that was part of a previous observational study, depression, quality of life, anxious symptoms and daytime sleepiness before and after CBD treatment.	Epilepsy and Behavior
Li et al., 2024	United Kingdom	UK Medical Cannabis Registry: a case series analyzing clinical outcomes of medical cannabis therapy for generalized anxiety disorder patients	To analyze key changes in anxiety-specific and general HRQL outcomes and safety in patients with GAD prescribed a homogenous selection of MCs, to reduce the associated biases that otherwise affect UK Medical Cannabis Registry data.	International Clinical Psychopharmacology
Barre et al., 2024	France	Limited Impact of Cannabidiol on Health-related Quality of Life of People with Long-term Controlled HIV: A Double-blind, Randomized, Controlled Trial	To test whether oral CBD-rich medication could significantly improve people with HIV's HRQL.	Open Forum Infectious Diseases
Lent et al., 2024	United States	Changes in health-related quality of life over the first three months of medical marijuana use	To evaluate changes in eight domains of HRQL over the first three months of medical marijuana treatment.	Journal of Cannabis Research
Dujic et al., 2024	Croatia	Chronic Cannabidiol Administration Mitigates Excessive Daytime Sleepiness and Fatigue in Patients with Primary Hypertension: Insights from a Randomized Crossover Trial	To establish whether chronic CBD supplementation will improve self-reported outcomes related to quality of life.	Cannabis and Cannabinoid Research
Sridharan et al., 2024	United Kingdom	Comparison of Cannabis-Based Medicinal Product Formulations for Fibromyalgia: A Cohort Study	To analyze changes in general health-related quality of life (HRQoL) and fibromyalgia symptom severity assessed by PROMs.	Journal of Pain and Palliative Care Pharmacotherapy
Haupts et al., 2024	Germany	Patient-reported benefits from nabiximols treatment in multiple sclerosis-related spasticity exceed conventional measures	To investigate the effectiveness of nabiximols in a real-world setting for the treatment of MS spasticity using GAS as a primary measure of treatment success.	Neurodegenerative Disease Management
Lynskey et al., 2024	United Kingdom	Prescribed Medical Cannabis Use Among Older Individuals: Patient Characteristics and Improvements in Well-Being: Findings from T21	To describe the characteristics of older individuals (aged 65 years or older) seeking MCs in the UK, examine 3-month changes in measures of well-being (general health, quality of life, mood/depression and sleep) in this group and compare their characteristics and treatment outcomes with those in younger individuals.	Drugs and Aging
Brett et al., 2024	United States	An observational time-series study on the behavioral effects of adjunctive artisanal cannabidiol use by adults with treatment resistant epilepsies	To determine if independent adjunctive use of artisanal CBD was beneficial for adults with treatment resistant epilepsies.	BMC Neurology
Murphy et al., 2024	United Kingdom	Clinical outcome analysis of patients with multiple sclerosis - Analysis from the UK Medical Cannabis Registry	To assess changes in MS-specific and general HRQL outcomes alongside adverse event incidence in patients prescribed MCs for MS from the UK.	Multiple Sclerosis and Related Disorders
Vivek et al., 2024	United Kingdom	UK Medical Cannabis Registry: Assessment of clinical outcomes in patients with insomnia	To assess changes in sleep-specific HRQL for those prescribed cannabis-based medicinal products for insomnia.	Brain and Behavior
Murphy et al., 2024	United Kingdom	A cohort study comparing the effects of medical cannabis for anxiety patients with and without comorbid sleep disturbance	To compare the PROMs of patients prescribed MCs for GAD, with and without impaired sleep	Neuropsychopharmacology Reports

1st Author, year	Country	Title	Study Aims	Journal
Aiewtrakoon et al., 2024	Thailand	Efficacy and Safety of Cannabidiol Oil on Chronic Insomnia: The First Randomized, Double-Blind, Placebo-Controlled, Crossover, Pilot Study in Thailand	To investigate the impact of a continuous four-week sublingual CBD intake on sleep indicators.	Journal of the Medical Association of Thailand
Kelley et al., 2024	United States	Observational Analysis of the Influence of Medical Marijuana Use on Quality of Life in Patients	To identify a potential relationship between cannabis use and quality of life measures including physical and emotional limitations, energy and fatigue, pain, and physical and social functioning.	Medical Cannabis and Cannabinoids
Francis et al., 2024	United Kingdom	Assessment of clinical outcomes in patients with inflammatory arthritis: Analysis from the UK Medical Cannabis Registry	To assess changes in validated PROMs after initiation of cannabis-based medicinal products and the safety of MC in patients with inflammatory arthritis.	International Clinical Psychopharmacology
Hershkovich et al., 2023	Israel	The role of cannabis in treatment-resistant fibromyalgia women.	To use the World Health Organization Quality of Life Bref questionnaire to characterize the impact of Cannabis Treatment initiation on the quality of life in women suffering from treatment-resistant fibromyalgia.	Pain practice
Vigano et al., 2023	Canada	The Quebec Cannabis Registry: Investigating the Safety and Effectiveness of Medical Cannabis.	To investigate the safety and effectiveness of medical cannabis in the real-world clinical practice setting.	Cannabis and cannabinoid research
Tait M, et al., 2023	Australia	Health-related quality of life in patients accessing medicinal cannabis in Australia: The QUEST initiative results of a 3-month follow-up observational study.	To assess overall HRQL, pain, fatigue, sleep, anxiety, and depression in a large real-world sample of patients accessing prescribed medicinal cannabis.	PloS one
Ergisi et al., 2023	United Kingdom	An Updated Analysis of Clinical Outcome Measures Across Patients From the UK Medical Cannabis Registry.	To analyze safety and the change in HRQL outcomes for all patients enrolled in the UKMCR who were prescribed MCs. Supplementary aims included evaluation of dosage regimens.	Cannabis and cannabinoid research
Arkell et al., 2023	Australia	Assessment of Medical Cannabis and Health-Related Quality of Life.	To assess whether patients using medical cannabis report improvements in health-related quality of life over time.	JAMA network open
Nathan et al., 2023	United States	Assessing Efficacy and Use Patterns of Medical Cannabis for Symptom Management in Elderly Cancer Patients.	To evaluate the effect of MC on the symptoms of this patient population. Secondary outcome measures include summary data on medical cannabis formulations and ratios used.	The American journal of hospice & palliative care
Hardy et al., 2023	Australia	Phase IIb Randomized, Placebo-Controlled, Dose-Escalating, Double-Blind Study of Cannabidiol Oil for the Relief of Symptoms in Advanced Cancer (MedCan1-CBD).	To assess whether CBD oil, when used in conjunction with standard palliative care, reduced symptom burden in patients with advanced cancer.	Journal of clinical oncology
Rifkin-Zybutz et al., 2023	United Kingdom	Clinical outcome data of anxiety patients treated with cannabis-based medicinal products in the United Kingdom: A cohort study from the UK medical cannabis registry.	To assess changes in anxiety symptoms as measured by the GAD-7 and the incidence of adverse events as collected by self-report form. The effect of type of MC product or previous cannabis experience influenced GAD-7 scores or the reporting of an adverse event was also analysed. The secondary aim was to investigate sleep quality and health-related quality of life outcomes.	Psychopharmacology
Tait J, et al., 2023	United Kingdom	Clinical outcome data of chronic pain patients treated with cannabis-based oils and dried flower from the UK Medical Cannabis Registry.	To evaluate changes in general HRQL, chronic pain-specific outcomes, and adverse events in patients treated exclusively with Cannabis-based medicinal products.	Expert Review of Neurotherapeutics

1st Author, year	Country	Title	Study Aims	Journal
Bapir et al., 2023	United Kingdom	Comparing the effects of medical cannabis for chronic pain patients with and without co-morbid anxiety: A cohort study.	To compare the outcomes of chronic pain patients with and without co-morbid anxiety following MC treatment.	Expert Review of Neurotherapeutics
Nicholas et al., 2023	United Kingdom	UK medical cannabis registry: Assessment of clinical outcomes in patients with headache disorders.	To assess changes in validated PROMs.	Expert Review of Neurotherapeutics
Vickery et al., 2022	Australia	A large Australian longitudinal cohort registry demonstrates sustained safety and efficacy of oral medicinal cannabis for at least two years.	To analyse collected Patient MC dose, safety and validated outcome data.	PloS one
Aungsumart et al., 2021	Thailand	A pilot study of the government pharmaceutical organization (GPO) cannabis extract for multiple sclerosis (MS) spasticity treatment in Thailand	To evaluate the efficacy and safety of Government Pharmaceutical Organization cannabis extract in the treatment of spasticity in MS patients in Thailand.	Journal of the Medical Association of Thailand
Eibach et al., 2021	Germany	Cannabidivarin for HIV-Associated Neuropathic Pain: A Randomized, Blinded, Controlled Clinical Trial	To investigate cannabidivarin (CBDV) a novel phytocannabinoid derived from the Cannabis sativa L. plant, in patients with HIV-associated neuropathic pain.	Clinical Pharmacology & Therapeutics
Erridge et al., 2021	United Kingdom	An initial analysis of the UK Medical Cannabis Registry: Outcomes analysis of first 129 patients	To analyze the early outcomes of the first series of patients prescribed MCs in the UK with respect to effects on HRQL and clinical safety.	Neuropsychopharmacology Reports
Gruber et al., 2021	United States	No pain, all gain? Interim analyses from a longitudinal, observational study examining the impact of medical cannabis treatment on chronic pain and related symptoms	To examine patients using MC to treat chronic pain pre versus post MC treatment.	Experimental and Clinical Psychopharmacology
Kawka et al., 2021	United Kingdom	Clinical Outcome Data of First Cohort of Chronic Pain Patients Treated With Cannabis-Based Sublingual Oils in the United Kingdom: Analysis From the UK Medical Cannabis Registry	To investigate the HRQL outcomes of patients with chronic pain who were prescribed MC oil preparations.	Journal of Clinical Pharmacology
Lucas et al., 2021	Canada	Cannabis Significantly Reduces the Use of Prescription Opioids and Improves Quality of Life in Authorized Patients: Results of a Large Prospective Study	To identify the primary characteristics of a large national cohort of medical cannabis patients in Canada, including detailed patterns of cannabis use; to assess the impacts of regulated, physician-supervised MC access on prescription substance use and quality of life; and to analyze key variables potentially associated with changes in prescription drug use and quality of life over 6 months.	Pain Medicine
Meng et al., 2021	Canada	Patient-reported outcomes in those consuming medical cannabis: a prospective longitudinal observational study in chronic pain patients	To assess the impact of cannabis on pain intensity and pain-related interference for patients consuming cannabis for up to 12 months. Secondary aims were to identify associated changes in opioid consumption, mental health, quality of life, and general health symptoms.	Canadian Journal of Anesthesia
Naftali et al., 2021	Israel	Cannabis is associated with clinical but not endoscopic remission in ulcerative colitis: A randomized controlled trial	To assess the effect of cannabis in improving clinical and inflammatory outcomes in ulcerative colitis patients.	PLoS ONE
Peterson et al., 2021	United States	Measuring the Change in Health-Related Quality of Life in Patients Using Marijuana for Pain Relief	To determine if there is a relationship between HRQL and MC use in patients using it to relieve pain.	Medical Cannabis and Cannabinoids

1st Author, year	Country	Title	Study Aims	Journal
Schloss et al., 2021	Australia	A Phase et al., 2 Randomised Clinical Trial Assessing the Tolerability of Two Different Ratios of Medicinal Cannabis in Patients With High Grade Gliomas	To investigate the tolerability of two different ratios of oral medicinal cannabis oil in patients who have been diagnosed with high grade gliomas as an adjunct to standard treatment.	Frontiers in Oncology
Naftali et al., 2021	Israel	Oral CBD-rich Cannabis Induces Clinical but Not Endoscopic Response in Patients with Crohn's Disease, a Randomised Controlled Trial	To evaluate efficacy of oral use of cannabis oil rich in CBD for induction of clinical, laboratory, and endoscopic remission in mild-to-moderate Crohn's disease.	Journal of Crohn's & colitis
Capano et al., 2020	United States	Evaluation of the effects of CBD hemp extract on opioid use and quality of life indicators in chronic pain patients: a prospective cohort study	To investigate the impact of full hemp extract cannabidiol (CBD) on opioid use and quality of life indicators among chronic pain patients.	Postgraduate Medicine
Chaves et al., 2020	Brazil	Ingestion of a THC-Rich Cannabis Oil in People with Fibromyalgia: A Randomized, Double-Blind, Placebo-Controlled Clinical Trial	To determine the benefit of a tetrahydrocannabinol (THC)-rich cannabis oil on symptoms and quality of life of fibromyalgia patients.	Pain Medicine
Gulbransen et al., 2020	New Zealand	Cannabidiol prescription in clinical practice: An audit on the first 400 patients in New Zealand	To review the changes in self-reported quality of life measurements, drug tolerability, and dose-dependent relationships in patients prescribed CBD oil for various conditions at a single institution.	BJGP Open
Safakish et al., 2020	Canada	Medical cannabis for the management of pain and quality of life in chronic pain patients: A prospective observational study	To evaluate the short-, and long-term effects of plant-based MC on outcomes of interest related to pain, quality of life, tolerability, and opioid medication use in a large cohort of chronic pain patients using medical cannabis over the course of one year.	Pain Medicine
Bar-Sela et al., 2019	Israel	Cannabis-related cognitive impairment: a prospective evaluation of possible influences on patients with cancer during chemotherapy treatment as a pilot study	To evaluate the effect of cannabis consumption on cognitive abilities as well as on symptom relief in patients with cancer during chemotherapy treatment.	Anti-Cancer Drugs
Gaston et al., 2019	United States	Quality of life in adults enrolled in an open-label study of cannabidiol (CBD) for treatment-resistant epilepsy	To compare QOL at baseline and after 1 year of treatment with CBD.	Epilepsy and Behavior
Palmieri et al., 2019	Italy	Spontaneous, anecdotal, retrospective, open-label study on the efficacy, safety and tolerability of cannabis galenical preparation (Bedrocan)	To investigate the short-term therapeutic effects, safety/tolerability and potential side effects of the cannabis galenical preparation (Bedrocan) in patients with a range of chronic conditions unresponsive to other treatments.	International Journal of Pharmacy Practice
Ueberall et al., 2019	Germany	Effectiveness and tolerability of THC:CBD oromucosal spray as add-on measure in patients with severe chronic pain: Analysis of 12-week open-label real-world data provided by the German pain e-registry	To assess 1)analgesic effects by using a composite responder definition that incorporates response- and relief-rates of a combination of nine different patient-reported/relevant parameters (such as pain intensity, pain-related disabilities with respect to daily life activities, sleep, overall well-being, physical and mental quality-of-life, depression, anxiety and stress),and 2) to evaluate prevalence and spectrum of treatment-emergent adverse events in response to MC as add-on treatment to pre-existing analgesic medications.	Journal of Pain Research
Markova et al., 2019	Czech Republic	Sativex as add-on therapy vs. further optimized first-line ANTispastics (SAVANT) in resistant multiple sclerosis spasticity: a double-blind, placebo-controlled randomised clinical trial	To evaluate the efficacy of MC oromucosal spray (Sativex) as add-on therapy to optimised standard antispasticity treatment in patients with moderate to severe MS spasticity.	International Journal of Neuroscience

1st Author, year	Country	Title	Study Aims	Journal
Irving et al., 2018	United Kingdom	A Randomized, Double-blind, Placebo-controlled, Parallel-group, Pilot Study of Cannabidiol-rich Botanical Extract in the Symptomatic Treatment of Ulcerative Colitis	To investigate the efficacy and safety of MC, CBD-rich botanical extract treatment, in patients with mild to moderate Ulcerative Colitis which had proved refractory to 5-ASA therapy.	Inflammatory Bowel Diseases
Gruber et al., 2016	United States	Splendor in the grass? A pilot study assessing the impact of medical marijuana on executive function	To assess the impact of 3 months of MC treatment on executive function, exploring whether MC patients would experience improvement in cognitive functioning, perhaps related to primary symptom alleviation.	Frontiers in Pharmacology
Haroutounian et al., 2016	Israel	The Effect of Medicinal Cannabis on Pain and Quality-of-Life Outcomes in Chronic Pain: A Prospective Open-label Study	To determine the long-term effect of MC treatment on pain and functional outcomes in participants with treatment-resistant chronic pain.	Clinical Journal of Pain
Haupts et al., 2016	Germany	Influence of Previous Failed Antispasticity Therapy on the Efficacy and Tolerability of THC:CBD Oromucosal Spray for Multiple Sclerosis Spasticity	To determine whether antispasticity treatment history influenced the efficacy and safety of add-on MC oromucosal spray in MS spasticity patients.	European Neurology
Russo et al., 2016	Italy	Should we care about sativex-induced neurobehavioral effects? A 6-month follow-up study	To characterize effects of 1- and 6-month Sativex administration in cannabis-naive MS patients including neurobehavioral function, tolerability, possible abuse, and effects on QoL and motor functions.	European Review for Medical and Pharmacological Sciences
Russo et al., 2016	Italy	Evaluating Sativex R in Neuropathic Pain Management: A Clinical and Neurophysiological Assessment in Multiple Sclerosis	To investigate the role of Sativex in improving pain in MSs patients.	Pain Medicine
Vermersch et al., 2016	Italy	Tetrahydrocannabinol: Cannabidiol oromucosal spray for multiple sclerosis-related resistant spasticity in daily practice	To collect data from everyday clinical practice concerning the effectiveness and tolerability of MC.	European Neurology
Ware et al., 2015	Canada	Cannabis for the Management of Pain: Assessment of Safety Study (COMPASS)	To evaluate safety issues in patients with chronic pain using cannabis as part of their pain management regimen.	Journal of Pain

CBD, cannabidiol; GAD, generalised anxiety disorder; HRQL, Health-related quality of life; IBD, inflammatory bowel disease; MC, Medicinal Cannabis; MS, Multiple Sclerosis; PROM, patient reported outcome measure; PTSD, Post-Traumatic Stress Disorder; QOL, Quality of Life; RCT randomised controlled trial; THC, tetrahydrocannabinol; UKMCR, UK Medical Cannabis Registry
