# Supplementary material for: Health-related quality of life in patients receiving medicinal cannabis: systematic review and meta-analysis of primary research findings 2015–2025
Source: Qual Life Res. 2026 Feb 1;35(3):56. doi: 10.1007/s11136-026-04170-7 (PMC12862010; doi:10.1007/s11136-026-04170-7)
Supplement: Supplementary file 4 — Supplementary Material 4 [file 11136_2026_4170_MOESM4_ESM.pdf]

Health-related quality of life in patients receiving medicinal cannabis: Systematic review and meta-analysis of primary research findings 2015 – 2025

Quality of Life Research

*Margaret-Ann Tait,[1,2,3], Louise Acret,[1,2,3] Daniel SJ Costa,[4] Kate White,[1,2,3] Rachel Campbell,[4] Claudia Rutherford[1,2,3]

[1]Susan Wakil School of Nursing, Faculty of Medicine and Health, University of Sydney, NSW, Australia
[2]Sydney Local Health District, NSW, Australia
[3]The Daffodil Centre, The University of Sydney, and Cancer Council NSW
[4]School of Psychology, Faculty of Science, University of Sydney, NSW, Australia
* margaret-ann.tait@sydney.edu.au

Online Resource 4: Health conditions of participants treated with Medicinal Cannabis (MC) in included studies.

	Health conditions of participants treated with MC	Number of studies recruiting participants with health condition
1	Agoraphobia	3
2	Alcohol use disorder	1
3	Alzheimer syndrome	3
4	Amyotrophic lateral sclerosis	1
5	Anorexia and wasting	6
6	Anxiety disorder	16
7	Arthritis / Osteoarthritis	7
8	Attention deficit / hyperactivity disorder	6
9	Autism	6
10	Back pain	4
11	Behavioral disorder	2
12	Benzodiazepine dependence	2
13	Bipolar disorder	2
14	Brain injury	2
15	Cancer symptoms	16
16	Cardiovascular disease	1
17	Chemotherapy-induced nausea and vomiting	5
18	Chronic fatigue	2
19	Chronic gum disease	1
20	Chronic kidney disease	2
21	Chronic neuropathic pain	9
22	Chronic pain	24
23	Complex regional pain syndrome	6
24	Congenital Adrenal Hyperplasia	1
25	Dementia	2
26	Depression	11
27	Diabetes	2
28	Dyslipidemia	1
29	Dysmenorrhea	1
30	Dystonia	2
31	Eating disorder	3
32	Ehlers-Danlos	3
33	Endometriosis	2
34	Epilepsy	13
35	Fibromyalgia	8
36	Gastrointestinal disorder	3
37	Glaucoma / intraocular eye pressure	4
38	Hashimoto's thyroiditis	1
39	Hereditary spastic paraplegia	2
40	HIV	2
41	Hypertension	2

42	Inflammatory Bowel Disease (ulcerative colitis and Crohn's disease)	14
43	Irritable bladder syndrome	1
44	Irritable bowel syndrome	3
45	Memory loss	1
46	Migraine/headache	11
47	Motor neuron disease	2
48	Movement disorder	1
49	Multiple Sclerosis	15
50	Multiple system atrophy	1
51	Narcolepsy	1
52	Nystagmus	1
53	Obsessive compulsive disorder	5
54	Opioid use disorder	2
55	Osteomyelitis	1
56	Palliative Care	3
57	Panic disorder	3
58	Parkinson disease	7
59	Perimenopause	1
60	Polymyalgia rheumatica	1
61	Posttraumatic stress disorder	12
62	Premenstrual dysphoric disorder	1
63	Psychiatric disorders	1
64	Refractory nausea and vomiting	6
65	Restless legs syndrome	1
66	Severe eczema	1
67	Sleep disorder / insomnia	12
68	Spasticity	4
69	Stress	1
70	Stroke	2
71	Substance use disorder	1
72	Tic disorder	2
73	Tinnitus	2
74	Topical steroid withdrawal syndrome	1
75	Tourette syndrome	3
76	Tremors	6
77	Vaginismus	1
