# Supplementary material for: Health-related quality of life in patients receiving medicinal cannabis: systematic review and meta-analysis of primary research findings 2015–2025
Source: Qual Life Res. 2026 Feb 1;35(3):56. doi: 10.1007/s11136-026-04170-7 (PMC12862010; doi:10.1007/s11136-026-04170-7)

Health-related quality of life in patients receiving medicinal cannabis: Systematic review and meta-analysis of primary research findings 2015 – 2025

Quality of Life Research
*Margaret-Ann Tait,[1,2,3], Louise Acret,[1,2,3] Daniel SJ Costa,[4] Kate White,[1,2,3] Rachel Campbell,[4] Claudia Rutherford[1,2,3]
[1]Susan Wakil School of Nursing, Faculty of Medicine and Health, University of Sydney, NSW, Australia
[2]Sydney Local Health District, NSW, Australia
[3]The Daffodil Centre, The University of Sydney, a joint venture with Cancer Council NSW
[4]School of Psychology, Faculty of Science, University of Sydney, NSW, Australia
* margaret-ann.tait@sydney.edu.au

Online Resource 6: Judgement on risk of bias (RoB 2) items for each included randomised controlled trial included in meta-analysis (n=8).

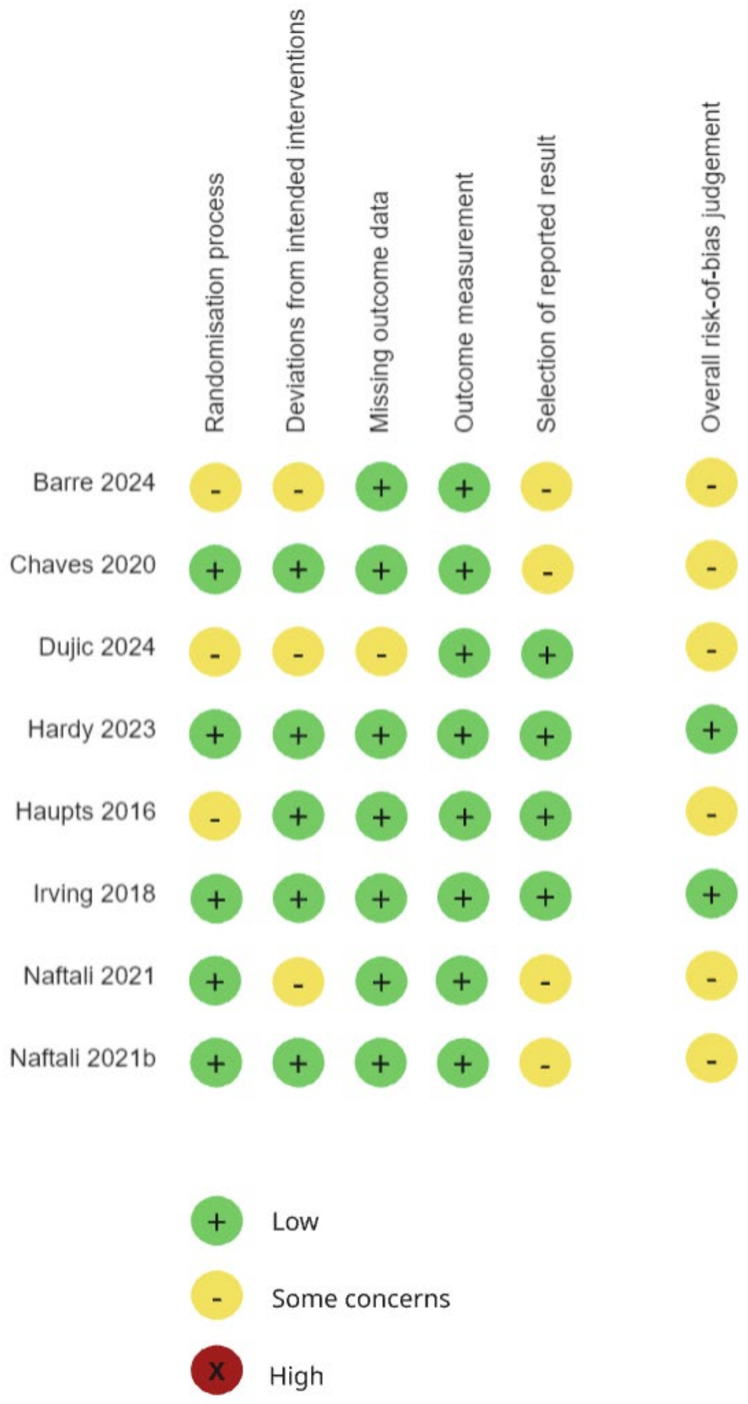

Supplement: Supplementary file 6 — Supplementary Material 6 [file 11136_2026_4170_MOESM6_ESM.pdf]
